# Supplementary material for: Olivine’s high radiative conductivity increases slab temperature by up to 200K
Source: Nat Commun. 2025 Jul 1;16:6058. doi: 10.1038/s41467-025-61148-8 (PMC12219095; doi:10.1038/s41467-025-61148-8)
Supplement: Supplementary file 1 — Supplementary Information [file 41467_2025_61148_MOESM1_ESM.pdf]

Supplementary Materials (SM) for

Olivine's high radiative conductivity increases slab temperature by up to 200 K

Enrico Marzotto[1,2*], Alexander Koptev[1], Sergio Speziale[1], Monika Koch Müller[1], Nada Abdel-Hak[1,3], Sarah B. Cichy[1,2], and Sergey S. Lobanov[1,2*]

[1]GFZ Helmholtz Centre for Geosciences, Telegrafenberg 14473 Potsdam, Germany

[2]Institute of Geosciences, University of Potsdam, Karl-Liebknecht-Straße 24-25, 14476 Potsdam, Germany

[3]Geology Department, Faculty of Science, Cairo University, 12613, Giza, Egypt

Corresponding authors: Enrico Marzotto (marzotto@gfz.de);
Sergey S. Lobanov (slobanov@gfz.de)

Methods

Sample synthesis. We followed the method described in Ref.[1]. A 1:1 mixture of Fe_2O_3 and SiO_2 powders was heated in a $Au_{80}Pd_{20}$ crucible to 1453 *K* for 24 h in a vertical gas mixing tube furnace at the Mineralogy Laboratory at the University of Potsdam. The desired oxygen fugacity of 10^{-12} units (below the magnetite-wüstite buffer[2]) was controlled by a gas mixture flux of CO_2 and Ar/H_2 (5% H_2), while continually monitoring the fugacity with a zircon lambda-sensor.

The use of fayalite as a spectroscopic model of mantle olivine. Standard preparation of the DAC sample cavity, such as used in this work, puts a constraint on the maximum sample thickness because of the starting diamond-to-diamond distance of ~40-50 μm. A sample of typical mantle olivine (Fe/Fe+Mg) ~0.1, with a thickness of 50 μm is still too thin to reliably detect the absorption coefficient of its crystal field bands. To circumvent this problem, we used fayalite as a spectroscopic model of mantle olivine. The appropriateness of this approach is illustrated on Fig. S1: the absorption coefficient at the Fe^{2+} crystal field bands at ~10000 cm^{-1} of San Carlos olivine and fayalite is proportional to the iron content. That is, the absorption coefficient of San Carlos olivine (Fa9.1) is approximately eleven times smaller than that of fayalite (Fa100). Note that only the γ-spectra are compared in Fig. S1 (i.e., polarized absorption spectra with electric field vector E parallel to the a-axis of olivine). The comparison of α- and β-spectra (E || b and E || c, respectively) for fayalite and San Carlos also showed that the absorption coefficient of the Fe^{2+} band scales near linearly with the Fe^{2+} content[3]. Please note also that the scaled absorption coefficient of fayalite is significantly higher at frequencies > 15000 cm^{-1}, possibly due to small amounts of Fe^{3+}, see Ref.[4]. This spectral range, however, is relatively unimportant for evaluating radiative conductivity at $T < 2000$ *K*, as is the case in this work, and was excluded from its evaluation. The above comparison shows that fayalite is an appropriate model of mantle olivine in the near-infrared spectral range, and we assume that fayalite remains an appropriate proxy of olivine also at high pressure. This assumption allowed us to scale the spectra of fayalite measured in LH-DAC experiments to represent mantle olivine by dividing the absorption coefficients by eleven to account for the lower Fe^{2+} content of mantle olivine compared to that of fayalite.

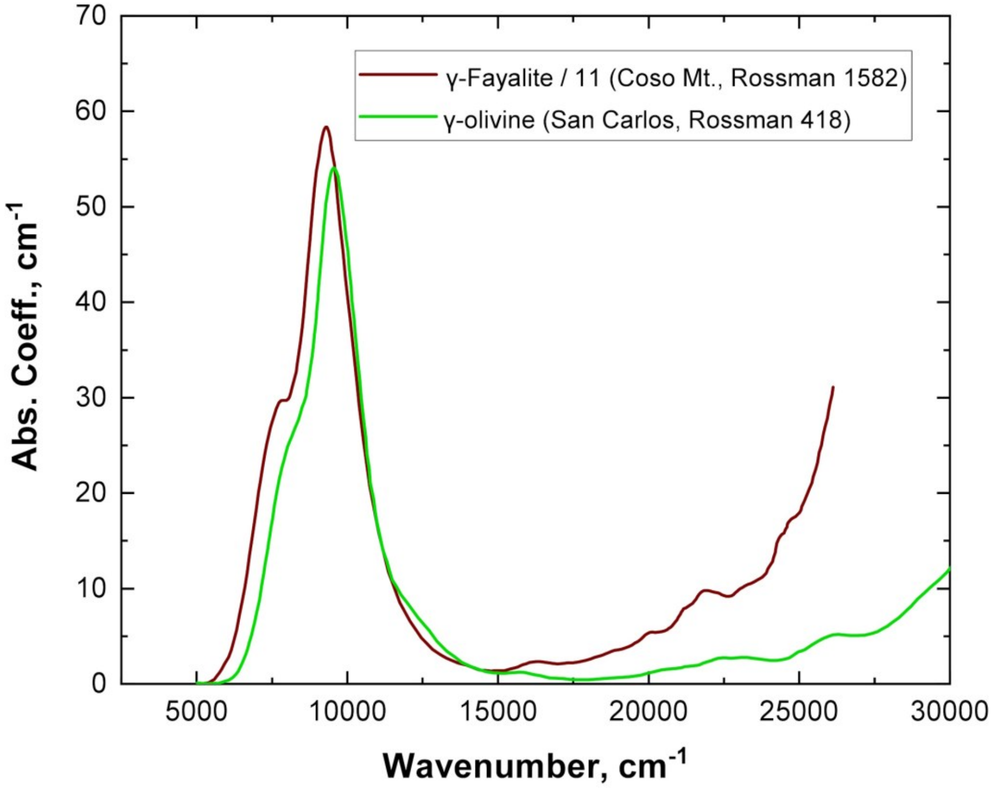

Fig. S1. Absorption coefficient of fayalite (#1582, Coso Mountains) and olivine (#418, San Carlos) measured along the γ-spectrum. The spectroscopic data is taken from the Mineral Spectroscopy Server (http://minerals.gps.caltech.edu/). The absorption coefficient of fayalite is divided by eleven to account for its correspondingly higher iron content. Note that the positions of the d-d bands in fayalite are ~300-500 cm^{-1} lower in energy than that in olivine because of the lowering of the crystal field splitting energy with increasing iron content.

HT-FTIR (LINKAM) experiments. The HT-FTIR (LINKAM) experiments were performed at 1 *atm* and up to 1273 *K* under Ar flow. Spectral data were collected every 100 *K* on heating and 200-300 *K* on cooling at ~2000-7000 cm^{-1} on a Bruker VERTEX 80V FTIR spectrometer equipped with KBr beamsplitter, HYPERION II microscope, and Mercury Cadmium Telluride detector. The temperatures recorded by the thermocouple were experimentally checked against the melting points of different salts and were in good agreement with the recordings. These measurements quantified the opacity of olivine in the infrared, which is quite discrepant across the extant literature[5-8], and is critical for evaluating Λ_{rad}^{Ol} because, at upper mantle temperatures (1000-2000 *K*) the peak of thermal emission is in the ~3500-7000 cm^{-1} spectral range, as described by the Wien's displacement law:

$$\lambda_{peak} = \frac{b}{T} \qquad \text{(S1)}$$

Where T is the absolute temperature, and b is Wien's displacement constant, equal to 2.898×10^{-3} $m{\cdot}K$.

LH-DAC experiments. The LH-DAC setup is a partially modified version of the setup reported previously[9]. The key feature of our system is the precise synchronization of ~1 *ns* supercontinuum probe pulses operating at 0.25 *MHz* to ~3 *ns* ICCD (intensified charge-coupled device, sensitive at > 11500 cm^{-1}) detector gates, which allows minimizing the thermal radiation seen by this detector. In this work, we upgraded this setup by installing a non-gated InGaAs 1D-array detector on the second output of the spectrograph (Princeton Instruments HRS-300). The InGaAs detector allows optical measurements in the ~6500-11500 cm^{-1} spectral range, which was critical for constraining temperature-induced changes in the optical absorbance of fayalite as well as for temperature measurements by radiometry at $T < \sim1200$ *K* (Fig. S2). The spectral sensitivity of our ICCD is mostly in the visible range, which allows reliable temperature measurements only at $T > 1200$ *K*. The spectral range above ~11500 cm^{-1} was recorded by the intensified-CCD (ICCD) detector of the original LH-DAC setup. The power of the 1064 *nm* heating laser was increased until the desired temperature was reached (first temperature measurement). Then, the supercontinuum probe intensity through the sample and thermal background were measured at high T by the ICCD and InGaAs detectors with ~600 *ns* and 2 *ms* exposures, respectively. No thermal background was detectable by the ICCD

while the 2 *ms* exposure of the InGaAs detector to fayalite at ~1000 *K* produced a notable but very reproducible thermal background that we subtracted from the supercontinuum probe transmission signal. Finally, the temperature measurement was repeated after the optical absorption measurements at high *T* to ensure its stability over the probing cycle (second temperature measurement). The agreement between the two temperature readings was typically within 10-20 *K*. Their average was assumed as the sample temperature at the time of the absorbance measurements. We also collected optical absorption spectra at room-temperature before and after each heating in order to test for reversibility over the heating cycles. Only fully reversible measurements were used to infer temperature-induced changes in fayalite opacity at high pressure. For example, in Fig. 1 (main text) are reported eight overlapping room-temperature spectra taken before and after the four high-temperature spectra.

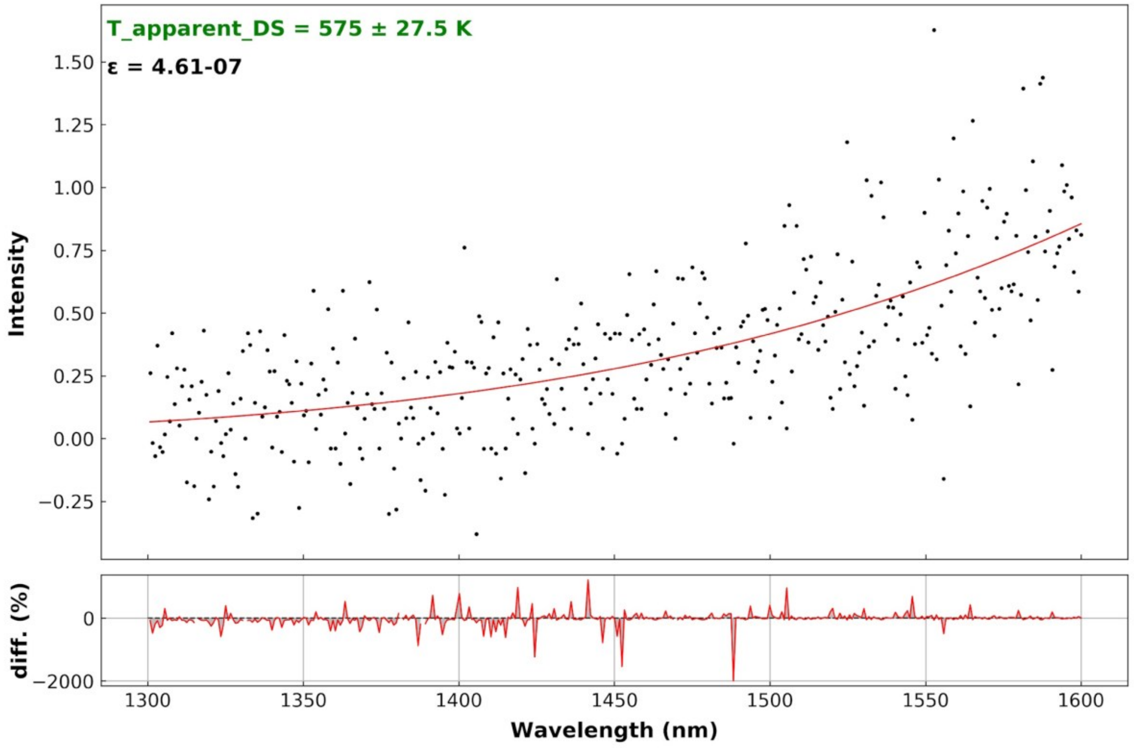

Fig. S2 | Representative thermal radiation spectrum collected in a laser-heated diamond anvil cell experiment with an InGaAs detector. Emissivity ε is obtained by fitting the thermal radiation spectrum with the blackbody model under the assumption that emissivity is wavelength independent

LH-DAC experiments offered insights into the effect of pressure on the absorption coefficient of olivine and provided verification of our LINKAM data in the overlapping spectral range.

Both types of experiments yielded the optical absorbance of the sample, which we converted into the absorption coefficient after an appropriate estimate of sample thickness. The absorption coefficients measured in the LH-DAC were extrapolated from ~6000 cm^{-1} down to ~2000 cm^{-1} using the temperature-derivatives inferred from LINKAM experiments and the data reported by Ref.[5].

Sample thickness and the absorption coefficient. In both types of experiments (LINKAM, LH-DAC), the absorption coefficient was evaluated as:

$$\alpha = -ln\,(10)\frac{1}{d}log_{10}(\frac{I_{sample}}{I_{reference}}) \quad \text{(S2)}$$

where d is the sample thickness I_{sample} is the probe intensity recorded through the sample (Fa9.1, Fa100), and $I_{reference}$ is the probe intensity recorded through the optical reference (Al_2O_3 plate for LINKAM, and NaCl pressure medium for LH-DAC), after appropriate background subtraction. Optical data were recorded on heating and cooling as a test for reproducibility (Fig. 1). The sample thickness in the LINKAM experiment was measured by a micrometer with an accuracy of ~ 1%. In LH-DAC experiments, all the spectra were measured on decompression, which allow minimizing non-isotropic changes in sample thickness in response to the uniaxial loading in the DAC[10]. The thickness of the fully decompressed Fa100 was measured directly by cutting through the sample with a focused ion beam and imaging the cut in a scanning electron microscope. The Fa100 thickness at high pressure was then obtained by assuming isotropic expansion upon decompression using an appropriate equation of state[11]. The error in sample thickness in LH-DAC experiments is ~10%.

Main results of the LH-DAC experiment. Fig. S3 shows the temperature-derivatives of the absorption coefficient of fayalite at 1.1. *GPa* (Fig. 1) after dividing them by eleven (to enable comparison to mantle olivine). As discussed above, the division by eleven is necessary to compare the fayalite absorption data to that of mantle olivine, which has a factor of eleven smaller iron content[3]. The agreement with the results of Ref.[7] is fairly good, considering that we only probe a non-oriented sample with a non-polarized light. The temperature-derivatives inferred from the LH-DAC experiment are also in good agreement with those measured in the LINKAM experiment in the overlapping spectral range (Fig. S4).

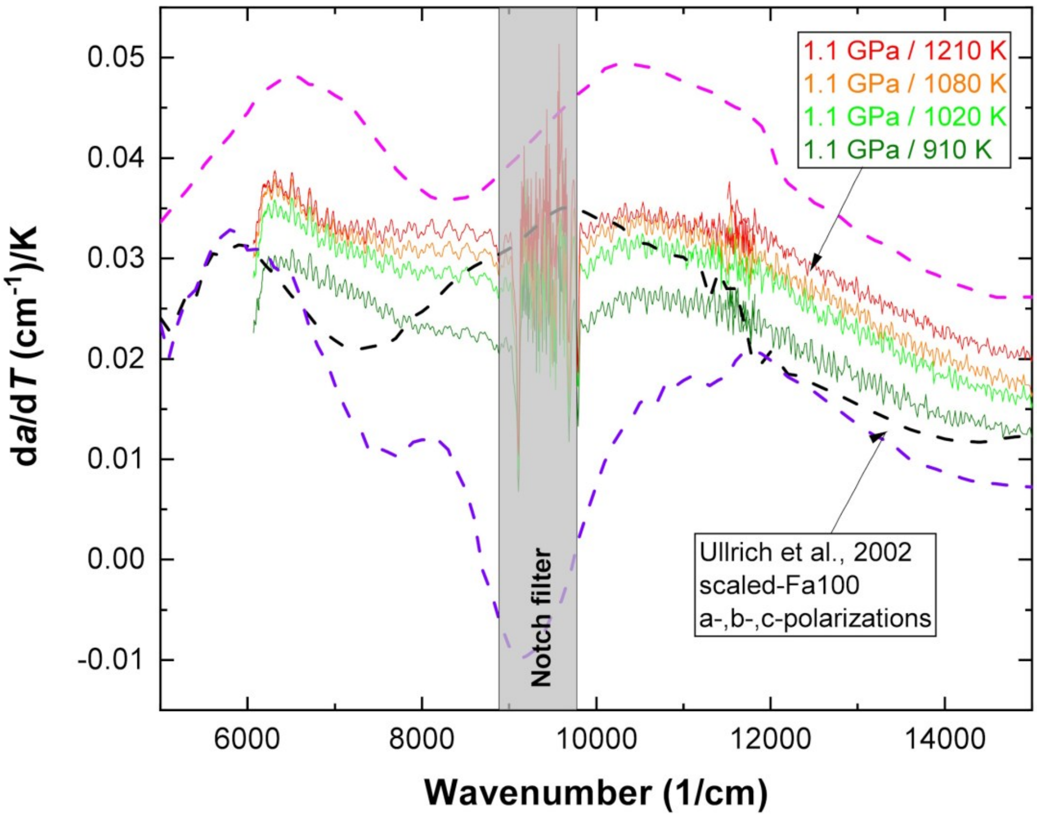

Fig. S3 | Temperature-derivatives of the absorption coefficient of fayalite at 1.1 *GPa* derived from absorption measured in the LH-DAC experiment (Fig. 1).

Main results of the LINKAM experiment. Fig. S4-left shows the measured absorption coefficients of Fa9.1 at 1 *atm*. The absorption coefficient increases with heating on the low and high-frequency ends of the plotted spectral range (*i.e.,* at ~2000-3000 cm^{-1} and 6000-7000 cm^{-1}) albeit not fully reversibly. Light absorption at ~2000-3000 cm^{-1} is governed by the fundamental lattice vibrations of olivine at <2000 cm^{-1} that broaden with temperature. Attenuation at 6000-7000 cm^{-1} is determined by the crystal field bands of Fe^{2+} in olivine (*i.e., d-d* electronic transitions). Qualitatively similar behavior has been recorded for olivine in previous experimental studies[5,7,12]. Important differences, however, emerge when comparing the temperature-derivatives of the absorption coefficient in this spectral range (Fig. S4-Right). Specifically, we find that our results agree best with the results reported by Ref.[5], in the overlapping temperature range (T < 1273 K). At high temperatures, which we could not access

in the LINKAM experiment, the temperature-derivatives extracted from Ref.[5] continue to increase with temperature (orange and red lines), independent of the probed crystal orientation. This behavior is also consistent with the spectra reported by Ref.[7], although frequencies below 5000 cm^{-1} were not probed in that work. Our results, however, are in sharp disagreement with the temperature-derivative extracted from the spectra reported by Ref.[8], whose high-temperature spectroscopic model of olivine at these frequencies is based on the measurements by Ref.[12] only up to 673 *K*. Specifically, the low frequency range does not show a strong increase in absorbance compared to what we find in our LIMKAM experiments and to the spectra reported by Ref.[5]. This is no small difference because this spectral range strongly contributes to the efficiency of radiative transport at upper mantle temperatures. Consequently, olivine radiative conductivity models based on Ref.[12] data overestimate the radiative contribution to its total thermal conductivity.

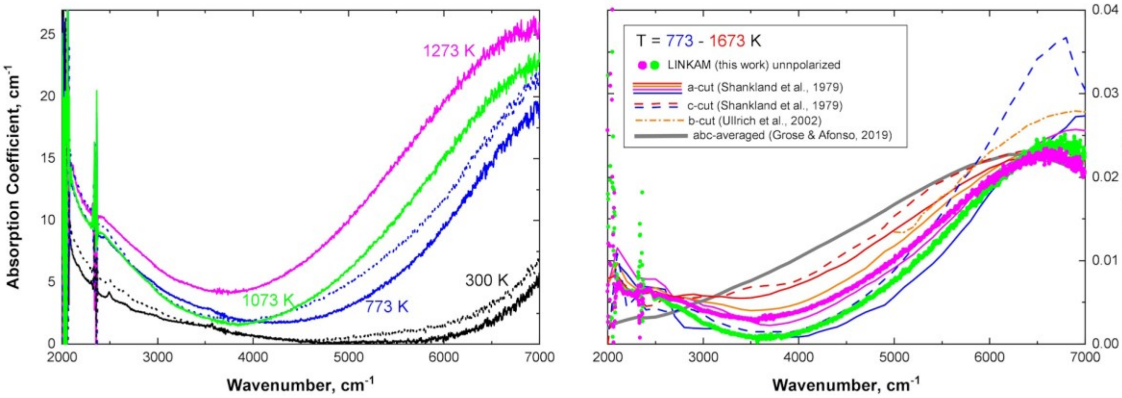

Fig. S4 | (LEFT) The absorption coefficient of Fa9.1 probed at 1 *atm* in the LINKAM experiment upon heating (solid spectra) and cooling (dotted spectra). (RIGHT) The temperature-derivatives of the absorption coefficient of Fa9.1 at 1 *atm* derived here upon heating (green and magenta circles) compared to that extracted from the spectra reported in the literature (solid and dashed curves). The color roughly codes the temperature range used to infer the temperature-derivative (grey: 673-300 *K*; blue: 773-300 *K*; orange: ~1350-300 *K*; red: 1673-300 *K*; magenta: 1273-300 *K*; green: 1073-300 *K*).

Modelling the optical absorption coefficient of peridotitic olivine at upper mantle *P-T* conditions. The broad agreement in the values of derivatives at overlapping frequencies inferred from high- and room-pressure experiments performed by us justified stitching them together to obtain a continuum in wavenumber derivative for modelling high-temperature spectra of olivine at high pressure. This temperature-derivative was then used in combination with the direction-averaged room-temperature absorption coefficient of San Carlos olivine (#418) at 1 *atm* reported on the Mineral Spectroscopy Server (http://minerals.gps.caltech.edu/Files/Visible/olivine/Index.html). The crystal-field bands of the absorption coefficient of San Carlos olivine at 1 *atm* were blue-shifted to account for their pressure-induced shift of ~120-160 cm^{-1}/GPa[4]. We ignored the pressure-induced shift of the absorption bands due to lattice vibrations (~1-4 cm^{-1}/GPa) because it is much smaller than that typical of crystal field bands. We did account, however, how iron content modifies the bands' frequencies thus affecting the temperature-derivative of fayalite absorption coefficient inferred in LH-DAC experiments because the crystal field splitting energy, and therefore the spectral position of *d-d* bands, is sensitive to iron content (e.g., Fig. S1). To this end we used the absorption coefficients of San Carlos olivine and Fayalite reported at https://doi.org/10.7907/jywr-qq57. Overall, by considering the temperature-, pressure-, and composition-dependences of the absorption coefficient and its temperature-derivative we were able to model the absorption coefficient of peridotitic olivine at ~1, 5, and 10 *GPa* and up to temperatures of 400-2000 *K*. These served as input into the calculation of the radiative thermal conductivity described below.

Calculation of radiative thermal conductivity from optical absorption coefficient. The radiative thermal conductivity shown in Fig. S5 was evaluated using the modelled high *P-T* absorption coefficients of olivine. Our radiative thermal conductivity data (Extended Data Tab. 1) were computed with the equation used in Ref.[13]:

$$\Lambda_{rad} = \frac{16n^2\sigma T^3}{3\alpha_r} \quad \text{(S3)}$$

where n is the refractive index of the medium, and σ is the Stefan-Boltzmann constant. The parameter α_r, instead, is the Rosseland (harmonic) mean absorption coefficient:

$$\frac{1}{\alpha_r} = \frac{\int_0^\infty \frac{1}{\alpha(\upsilon,T)} \frac{\delta I_{bb}(\upsilon,T)}{\delta T} \delta\upsilon}{\int_0^\infty \frac{\delta I_{bb}(\upsilon,T)}{\delta T} \delta\upsilon} \quad \text{(S4)}$$

The parameter $I_{bb}(\upsilon, T)$ is the Plank blackbody radiation.

$$I_{bb}(\upsilon,T) = \frac{2h\upsilon^3}{c^2}\left[exp\left(\frac{h\upsilon}{k_b T}\right) - 1\right]^{-1} \quad \text{(S5)}$$

where h is the Plank constant; c the speed of light, υ the frequency, k_b Boltzmann constant. The Rosseland absorption coefficient is the weighted average of the measured absorption coefficient $\alpha(\upsilon,T)$, whereas the weighting function $\delta I_{bb}(\upsilon,T)/\delta T$ is the temperature derivative of the Plank blackbody radiation. All constants used in the equations (S3)-(S5) are reported in Extended Data Tab. 3.

Uncertainties in radiative thermal conductivity estimates and its anisotropy. We estimate the relative error in olivine's radiative thermal conductivity Λ_{rad} at upper mantle conditions to be ~30%. The value of the relative error is primarily due to the uncertainty in sample thickness at high pressure and, to a smaller extent, to the uncertainty in temperature[9,10,14,15].

In this study, we measured the optical absorption coefficient of olivine in LH-DAC experiments using unpolarized light. Polarized optical properties of oriented olivine crystals can be found in the Aerosol Refractive Index Archive (ARIA) managed by the Earth Observation Data Group (EODG) at University of Oxford (https://eodg.atm.ox.ac.uk/ARIA/data?Minerals/Olivine). In the ARIA archive, are reported the olivine's optical properties along a, b, c directions collected by Ref.[16-18]. These data agree with our results and show that the intensity of absorption bands due to fundamental lattice vibrations is not a strong function of crystal orientation. Nevertheless, the frequency of some of the absorption bands does depend on the crystallographic orientation. This may result in orientation-dependent radiative conductivity at low temperature, but the T-induced broadening of these bands diminishes the anisotropy (compare Ref.[18] datasets at $T = 300\ K$ and at $T = 928\ K$). This inference is supported by Ref.[5] whose authors estimated the radiative conductivity

along the directions a and c and found that, at T > 1600 K, the difference is small (~10 %) (Fig 2, diamond markers).

Defining the *P*, *T*-dependent thermal conductivity equations for the numerical model. In the models, the temperature-dependent radiative thermal conductivity $\Lambda_{rad}(T)$ was computed using the following algorithm:

$$\begin{aligned} T &= T_{i,j}/T_n \\ x &= ln\,(T) \\ z &= m_1 x + c_0 \\ y_s &= \exp\,(z) \\ y' &= \frac{y_s}{(1+y_s)} \\ y &= y_{min} + (y_{max} - y_{min})y' \\ \Lambda_{rad}(T) &= \Lambda_{rad}^{n} \cdot exp\,(y) \end{aligned} \tag{S6}$$

Where $T_{i,j}$ indicates the temperature at a given node in the coordinate $[i,j]$, T its nondimensional value, normalized to $T_n = 1\ K$. The parameters m_1 and c_0 are coefficients for linear interpolation. The parameters y_{min} and y_{max} are the logarithms of the minimum and maximum values of the experimental dataset, normalized to a reference value ($\Lambda_{rad}^{n} = 1\ W\ m^{-1}\ K^{-1}$): (1) $y_{min} = \log(\Lambda_{rad}^{bot}/\Lambda_{rad}^{n})$, where Λ_{rad}^{bot} is the reference radiative thermal conductivity at room temperature ($T_0 = 298\ K$), here assumed to be equal to $10^{-10}\ W\ m^{-1}\ K^{-1}$; (2) $y_{max} = \log(\Lambda_{rad}^{top}/\Lambda_{rad}^{n})$, with $\Lambda_{rad}^{top} = \Lambda_{rad}^{max} + \sigma_{rad}$ where Λ_{rad}^{max} is the maximum value of the dataset and σ_{rad} its standard deviation. Note that the value $exp(y)$ is dimensionless; therefore, it is necessary to multiply the calculated value for the reference radiative thermal conductivity Λ_{rad}^{n} to assign the unit of measurement. The value of each coefficient (m_1, c_0, y_{min}, y_{max}) was extrapolated from literature data, and reported in Extended Data Tab. 1-2: olivine[This Study], wadsleyite[19], ringwoodite[19]. The Λ_{rad} profile reported in the main manuscript (see Fig. 2, red solid line) was computed using the algorithm (S6) and the adiabatic temperature profile[20]. Alternatively, $\Lambda_{rad}(T)$ can be computed with a more straightforward formulation by employing a 6th-order polynomial $\Lambda_{rad} = \sum_{i=0}^{6} b_i T^i$, extrapolated from the dataset reported in Fig. S5 and Extended Data Tab. 1. The algorithm (S6) employed in our numerical models, however, is bounded between minimum (Λ_{rad}^{bot}) and maximum values (Λ_{rad}^{top}), and is thus designed to self-consistently avoid, for any value of T, negative or unrealistic values of

Λ_{rad} which may compromise the solution of the heat diffusion equation (S27). We therefore recommend using the 6th-order polynomial to compute Λ_{rad}, only for the limited temperature range of 298 < T < 2000 K.

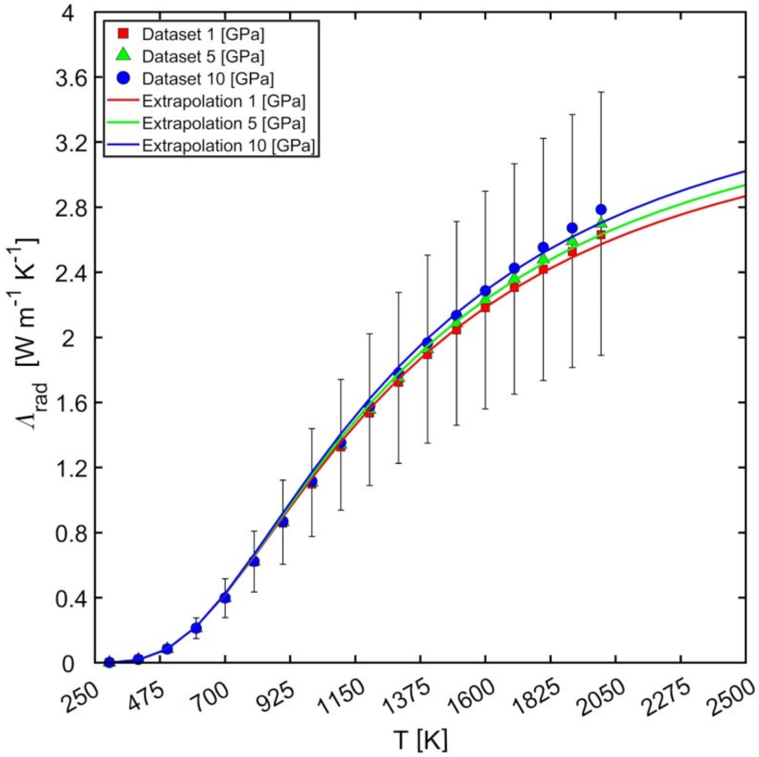

Fig. S5 | Radiative thermal conductivity Λ_{rad} of olivine (Fa9.1) as a function of temperature T $[K]$. The coloured markers indicate Λ_{rad} estimates derived from the absorption coefficient measurements at high P and high T. Each marker indicates a different dataset (see Extended Data Tab. 1): red squares 1.1 GPa; green triangles 5 GPa; blue circles 10 GPa. The solid lines indicate the radiative thermal conductivity computed, for each dataset, with algorithm (S6). The vertical bars represent ±30% uncertainty of the 5 GPa dataset. Alternatively, $\Lambda_{rad}(T)$ can also be computed by employing a 6th-order polynomial $\Lambda_{rad} = \sum_{i=0}^{6} b_i T^i$, using the coefficients reported in Extended Data Tab. 1.

In the models, the pressure-dependent lattice thermal conductivity Λ_{lat} *(P)* was computed using the following algorithm:

$$\begin{gathered} P = P_{i,j}/P_n \\ x = ln\,(P_{i,j}) \\ z = m_1 x + c_0 \\ y_s = \exp{(z)} \\ y' = \frac{y_s}{(1 + y_s)} \end{gathered} \tag{S7}$$

$$y = y_{min} + (y_{max} - y_{min})y'$$

$$\Lambda_{lat}(P) = \Lambda_{lat}^{n} \cdot exp\,(y)$$

Where P_{ij} indicates the temperature at a given node in the coordinate $[i,j]$, P its nondimensional value, normalized to $P_n = 1\ K$. The parameters m_1 and c_0 are coefficients for linear interpolation. The parameters y_{min} and y_{max} are the logarithms of the minimum and maximum values of the experimental dataset, normalized to a reference value (Λ_{lat}^{n} =1 $W\ m^{-1}\ K^{-1}$): (1) y_{min} = $\log(\Lambda_{lat}^{bot}/\Lambda_{lat}^{n})$, where Λ_{lat}^{bot} is the reference lattice thermal conductivity at room pressure (P_0 = 10^{-4} GPa); (2) $y_{max} = \log(\Lambda_{lat}^{top}/\Lambda_{lat}^{n})$, with $\Lambda_{lat}^{top} = \Lambda_{lat}^{max} + \sigma_{lat}$ where Λ_{lat}^{max} is the maximum value of the dataset and σ_{lat} its standard deviation. Note that the value $exp(y)$ is dimensionless; therefore, it is necessary to multiply the calculated value for the reference lattice thermal conductivity Λ_{lat}^{n} to assign the unit of measurement. The value of each coefficient (m_1, c_0, y_{min}, y_{max}) was extrapolated from Time-Domain Thermo-Reflectance (TDTR) datasets and reported in Extended Data Tab. 1-2: olivine[21], ringwoodite[22]. The relative error in TDTR lattice thermal conductivity measurements at upper mantle conditions is ~10-15%[21,22]. The P, T-dependent[23] lattice thermal conductivity $\Lambda_{lat}\ (P,\ T)$ was computed from $\Lambda_{lat}\ (P)$:

$$\Lambda_{lat}(P,T) = \Lambda_{lat}(P) * \left(\frac{T}{298}\right)^{a} \qquad \text{(S8)}$$

The factor $(T/298)^{a}$ modifies the lattice thermal conductivity as a function of temperature[23,24]. In our calculation we set the exponent a = -0.5[23]. The Λ_{lat} profile reported in the main manuscript (see Fig. 2, blue solid line) was computed using the algorithm (S7) and equation (S8) and the adiabatic temperature profile[20,25]. Total thermal conductivity was computed as:

$$\Lambda(P,T) = \Lambda_{lat}(P,T) + \Lambda_{rad}(T) \qquad \text{(S9)}$$

In the models, the aggregate thermal conductivity of the slab is equal to olivine Λ^{Ol} in the upper mantle (0-410 km), and ringwoodite Λ^{Rw} in the MTZ (410-660 km). The depths of the phase transitions are fixed using the values computed along the average mantle geotherm[20,25]. These values do not account for the effect of the slab temperature which causes upward or downward shift of the transition depth depending on the Clapeyron slope of the phase reaction[26]. The thermal conductivity profiles computed along the linear T gradient of the lithosphere

(35 K/km), and the adiabatic P, T profile of the mantle using eq. (S6)-(S9) are reported in Fig. S6.

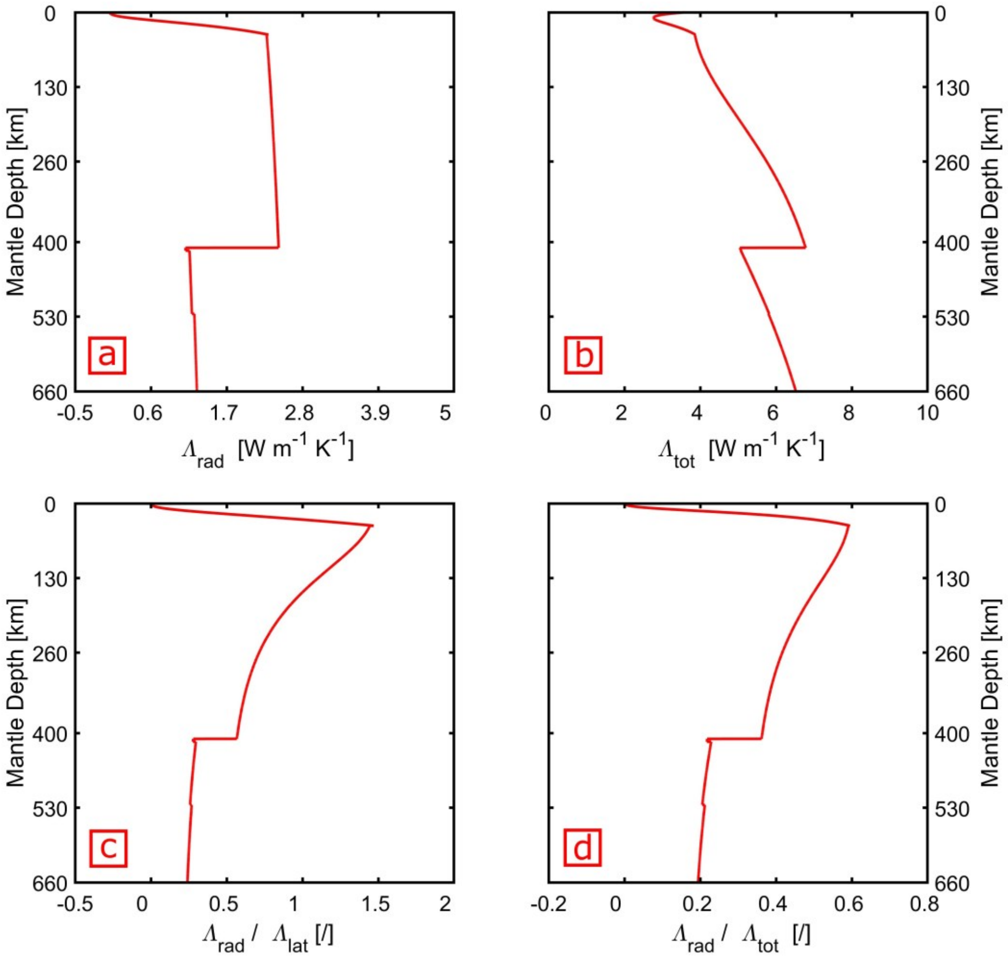

Fig. S6. | Thermal conductivity profiles from the surface down to the base of the Mantle Transition Zone (MTZ) at 660 *km* of depth. These profiles were computed along the linear *T* gradient of the lithosphere (35 *K/km*), and the adiabatic *P*, *T* profile of the mantle[20,25]. The subplots represents: a) radiative thermal conductivity Λ_{rad} profile computed using algorithm (S6); b) total thermal conductivity profile computed as $\Lambda_{tot} = \Lambda_{lat} + \Lambda_{rad}$ *;* where lattice conductivity Λ_{lat} was computed using algorithm (S7)-(S8); c) ratio between lattice and radiative components of thermal conductivity $\Lambda_{rad}/\Lambda_{lat}$; d) ratio between radiative and total thermal conductivity $\Lambda_{rad}/\Lambda_{tot}$.

Defining the thermal conductivity equations reported in literature. All Λ profiles reported in Fig.2 of the main manuscript were computed from the pressure gradient reported in the PREM model[25], the linear T gradient of the lithosphere (*35 K/km*), and the adiabatic temperature gradient reported in Ref.[20] of the mantle. Thermal conductivity profile #4 in the main manuscript (Fig. 2, dashed black line) was taken from Ref.[6] (*Hofmeister, Science, 1999*). The equation of $\Lambda_{Hof1999}$ includes the P, T-dependent lattice thermal conductivity and the T-dependent radiative thermal conductivity. The list of symbols and values is reported in Extended Data Tab. 3.

$$\Lambda_{Hof1999}(P,T) = \Lambda_0 \left(\frac{T}{298}\right)^a exp\left[-\left(4\gamma + \frac{1}{3}\right)\alpha(T-298)\right] \times \left(1 + \frac{K_0' P}{K_0}\right) + f \sum_{i=0}^{3} b_i T^i \tag{S10}$$

Thermal conductivity profile #5 in the main manuscript (see Fig. 2, dash-dotted red line) was taken from Ref.[12] (*Hofmeister, J. Geodyn., 2005*). The equation of $\Lambda_{Hof2005}$ computes aggregate radiative thermal conductivity of a rock as a function of T and a grain size d. The $\Lambda_{Hof2005}$ profile in Fig. 2 computed assuming a grain size of d = 1 *cm*.

$$\Lambda_{Hof2005}(d,T) = [5 + 22(1-d)]exp\left[\frac{-[T - 2800 - 2600(1-d)]^2}{[400 + 1400(1-d)]^2}\right] + [1.7 - 0.2(1-d)]exp\left[\frac{-[T - 1400 - 300(1-d)]^2}{[500 + 200(1-d)]^2}\right] \tag{S11}$$

Thermal conductivity profile #6 in the main manuscript (see Fig. 2, dashed crimson line) was taken from Ref.[8] (*Grose & Afonso, G-Cubed, 2019*), which elaborated an effective medium theory (EMT) to compute Λ_{rad} as a function of temperature and grain size. The $\Lambda_{G\&A2019}$ equation is a 6th-order polynomial, extracted from Fig. 6 of Ref.[8] with WebPlotDigitizer (https://apps.automeris.io/wpd/), to compute the T-dependent radiative thermal conductivity of a rock with a grain size of 1 *cm* (see Extended Data Tab. 3).

$$\Lambda_{G\&A2019} = \sum_{i=0}^{6} b_i T^i \tag{S12}$$

Thermal conductivity data #7 in the main manuscript (see Fig. 2, coral diamond markers) were taken from Ref.[5] (*Shankland et al., JGR: Solid Earth, 1979*), see Extended Data Tab. 3.

Effects of grain boundary scattering on thermal conductivity. Phonon mean-free-path l_p has a first order effect on the lattice thermal conductivity Λ_{lat}[27]. Similarly, radiative thermal conductivity Λ_{rad} is mainly governed by the spectral mean free path of photons[8]. Both lengths quantify the distance a thermal energy carrier (phonon and photon) can travel without being scattered i.e., changing direction, or losing energy. Scattering of energy carriers generate thermal resistance, thus reducing the total thermal conductivity[28].

Scattering is caused by the presence of: (a) crystallographic defects[29] (i.e. small scale interruptions in the crystal lattice, like vacancies or dislocations); (b) grain boundaries[28] (i.e., large-scale interruption of the crystal lattice); (c) the presence of heavy or light chemical impurities[27] (i.e., elements with large mass difference compared to neighbor atoms in the crystal lattice); and (d) temperature[29] (which increases the number of phonons propagating through the crystal lattice, thus increasing the probability of phonon-phonon interactions and consequent annihilations).

Typically, phonon mean free path is in the order of a few *nm*[27], whereas photon mean free path can vary considerably – from *nm* to *mm* - depending on the extinction coefficient *e* of the polycrystalline aggregate[8], which is the sum of the absorption and scattering coefficients. To first order, the scattering coefficient μ[30] is inversely proportional to the grain size $\sim 1/d$[29]. At low temperature, most of the phonon scattering occurs in the inter-grain regions (grain boundaries), where the crystallographic disorder concentrates[28]. This behavior has been observed in thermal conductivity measurements of polycrystalline aggregates of mica, where the anomalous decrease of Λ_{lat} for increasing P was attributed to the grain size reduction due to the crushing of the sample[31,32]. At high temperature, however, the frequent phonon-phonon interactions cause a drastic reduction of the phonon mean free path[29] so that the other mechanisms, such as defects and impurities, have negligible influence upon scattering[27]. Therefore - unless the grain size of the polycrystalline aggregate is less than the phonon mean free path (few *nm*) - grain boundary scattering at high temperature should have a minor impact on the lattice thermal conductivity[28].

Estimating the reduction of radiative thermal conductivity at the grain boundaries, however, is more challenging because it requires reliable estimates of the extinction coefficient μ[15,29] (i.e., the effective opacity of a polycrystal aggregate). Ref.[8] elaborated an effective medium theory (EMT) as a function of temperature and grain size. These theoretical calculations show that

grain boundary scattering becomes non-negligible for grain sizes < 1 mm (see Fig. 7 in Ref.[8]). For an isotropic upper mantle assemblage with a grain size of 100 μm, grain boundaries scattering reduces Λ_{rad} by ~25% for $T < 1500$ K, and by ~15% for $T \geq 1500$ K. The grain size of the upper mantle estimated from experimental constraints[33,34] and geophysical observation[35] is in the order of a few mm. This is consistent with the grain distribution of mantle xenolits[36] which spans 3 orders of magnitudes, from 200 μm to 25 mm. Therefore, the reduction of Λ_{rad} due to grain boundary scattering in a polycrystalline upper mantle aggregate should be at most ~25% in fine-grained portions of the mantle (e.g., cold shear zones), and negligible in the coarser portion of the mantle where the grain size is ≥1 mm (e.g., warm convective mantle). As the temperature inside the slab increases, annealing processes lead to grain growth[33], thus reducing grain boundary scattering. Therefore, for typical slab temperatures of $T > 1000$ K, the average grain size should be large enough to mitigate the effects of grain boundary scattering on both Λ_{lat} and Λ_{rad}. To summarize, grain-boundaries scattering could be relevant when modelling heat diffusion inside fine-grained shear zone in subducting slabs (see e.g., Ref.[37]) but for the goal of this manuscript (i.e. modelling heat diffusion in the bulk of a subducting slab) phonon and photon scattering at grain boundaries is insignificant and can be ignored. The effects of -30% Λ_{rad} on slab's thermal evolution have been tested in the lower uncertainty bound model Λ_{rad} (see Fig. S13), and gives ~50 K colder slabs compared to the $\Lambda_{rad} > 0$ reference case (Fig. 3.b).

Defining the *P*, *T*-dependent density and specific heat capacity for the numerical model. All the coefficients reported in this section are reported in Extended Data Tab. 4. *P*- and *T*-dependent densities were calculated from high-pressure unit cell volume V_{cell}^{x}[Å^3] datasets reported in literature: Ref.[38] for olivine $V_{cell}^{Ol}(P)$, and Ref.[39] for both wadsleyite and ringwoodite which were assumed to have the same unit cell volume $V_{cell}^{Wd}(P) = V_{cell}^{Rw}(P)$. From each dataset, we extrapolated the coefficients of a 4th-order polynomial:

$$V_{cell}^{x}(P) = a^{x}P^{4} + b^{x}P^{3} + c^{x}P^{2} + d^{x}P + V_{0}^{x} \tag{S13}$$

The high-*P* V_{cell}^{x} of a given mineral was then converted into molar volume V_{mol}^{x} $[cm^{3}\ mol^{-1}]$:

$$V_{mol}^{x}(P) = \frac{V_{cell}^{x}(P) \cdot 6.022}{Z^{x} * 10} \tag{S14}$$

Z^x represents the number of formulas per unit cell of a mineral ($Z^{Ol} = 4$; $Z^{Rw} = 4$), whereas 6.022 is the coefficient of the Avogadro's number ($N_A = 6.022 \times 10^{23}$ mol^{-1}). Room T density $\rho_0^x(P, T_0)$ of each mineral was computed using Mg_2SiO_4 molar weight $W_{mol}^{\mathrm{Mg2SiO4}} = 140.69\ g\ mol^{-1}$:

$$\rho_0^x(P, T_0) = \frac{W_{mol}^{Mg2SiO4}}{V_{mol}^x(P, T)} \cdot 1000 \tag{S15}$$

The factor 1000 in eq. (S15) is needed to convert $[g\ cm^{-3}]$ into $[kg\ m^{-3}]$. T-dependent density $\rho^x(P, T)$ was computed with the equation reported in Ref.[40]:

$$\rho^x(P, T) = \rho_0^x(P, T_0)\,(1 - \alpha^x(P, T) \cdot \Delta T) \tag{S16}$$

Where $\alpha^x(P, T)$ is P,T-dependent thermal expansion coefficient of the phase x. For olivine, $\alpha^{Ol}(P, T)$ was computed with the equation reported in Ref.[41]:

$$\alpha^{Ol}(P, T) = \alpha_0^{Ol} + e^{Ol}T + f^{Ol}P + g^{Ol}PT + h^{Ol}P^2 \tag{S17}$$

For wadsleyite, we assumed $\alpha^{Wd}(P, T) = \alpha^{Rw}(P, T)$, which was extrapolated from ringwoodite dataset reported in Ref.[42]:

$$\alpha^{Rw}(P, T) = Q_\alpha P^2 + L_\alpha P + I_\alpha \tag{S18}$$

where $Q_\alpha(T)$ is the T-dependent quadratic coefficient, $L_\alpha(T)$ is the T-dependent linear coefficient, and $I_\alpha(T)$ is the T-dependent intercept:

$$Q_\alpha(T) = i_Q^{Rw}T^4 + j_Q^{Rw}T^3 + k_Q^{Rw}T^2 + l_Q^{Rw}T + m_Q^{Rw} \tag{S19}$$

$$L_\alpha(T) = \mathrm{i}_L^{\mathrm{Rw}}T^4 + \mathrm{j}_L^{\mathrm{Rw}}T^3 + \mathrm{k}_{\mathrm{L}}^{\mathrm{Rw}}T^2 + \mathrm{l}_L^{\mathrm{Rw}}T + \mathrm{m}_L^{\mathrm{Rw}}$$

$$I_\alpha(T) = \mathrm{i}_I^{\mathrm{Rw}}T^4 + \mathrm{j}_I^{\mathrm{Rw}}T^3 + \mathrm{k}_I^{\mathrm{Rw}}T^2 + \mathrm{l}_I^{\mathrm{Rw}}T + \mathrm{m}_I^{\mathrm{Rw}}$$

Olivine's specific heat capacity Cp_{Sp}^{Ol} $[J\ kg^{-1}K^{-1}]$ was computed with the equation in Ref.[41]:

$$Cp_{Sp}^{Ol}(P, T) = Cp_0^{Ol} + n^{Ol}T^{-0.5} + o^{Ol}T^{-2} + p^{Ol}P + q^{Ol}P^2 \tag{S20}$$

Wadsleyite's and ringwoodite's specific heat capacity were assumed to be equal $Cp_{Sp}^{Wd}(P, T) = Cp_{Sp}^{Rw}(P, T)$, and computed from ringwoodite's molar heat capacity $Cp_{mol}^{Rw}[J\ mol^{-1}K^{-1}]$ which was extrapolated from Ref.[42] dataset:

$$Cp_{mol}^{Rw}(P, T) = Q_{Cp}P^2 + L_{Cp}P + I_{Cp} \tag{S21}$$

where $Q_{Cp}(T)$ is the temperature-dependent quadratic coefficient, $L_{Cp}(T)$ is the temperature-dependent linear coefficient, and $I_{Cp}(T)$ is the temperature-dependent intercept.

$$Q_{Cp}(T) = r_Q^{Rw}T^4 + s_Q^{Rw}T^3 + t_Q^{Rw}T^2 + u_Q^{Rw}T + v_Q^{Rw} \tag{S22}$$

$$L_{Cp}(T) = \mathrm{r}_L^{\mathrm{Rw}}T^4 + \mathrm{s}_L^{\mathrm{Rw}}T^3 + \mathrm{t}_L^{\mathrm{Rw}}T^2 + \mathrm{u}_L^{\mathrm{Rw}}T + \mathrm{v}_L^{\mathrm{Rw}}$$

$$I_{Cp}(T) = \mathrm{r}_I^{\mathrm{Rw}}T^4 + \mathrm{s}_I^{\mathrm{Rw}}T^3 + \mathrm{t}_I^{\mathrm{Rw}}T^2 + \mathrm{u}_I^{\mathrm{Rw}}T + \mathrm{v}_I^{\mathrm{Rw}}$$

Ringwoodite's specific heat capacity Cp_{Sp}^{Rw} was given by the relation:

$$Cp_{Sp}^{Rw}(P,T) = \frac{Cp_{mol}^{Rw}(P,T)}{W_{mol}^{Mg2SiO4}} \tag{S23}$$

Pressure and temperature dependent density *ρ(P,T)* and specific heat capacity *Cp(P,T)* are fundamental to computing the *P*,*T*-dependent heat diffusivity *κ(P,T)* [m^2 s^{-1}]:

$$\kappa(P,T) = \frac{\Lambda_{tot}(P,T)}{\rho(P,T)Cp(P,T)} \tag{S24}$$

In Fig. S7 are reported the radial profiles of the different thermodynamic parameters computed along the linear T gradient of the lithosphere (35 K/km) and the adiabatic P,T profile of the mantle[20,25] using eq. (S13)-(S24).

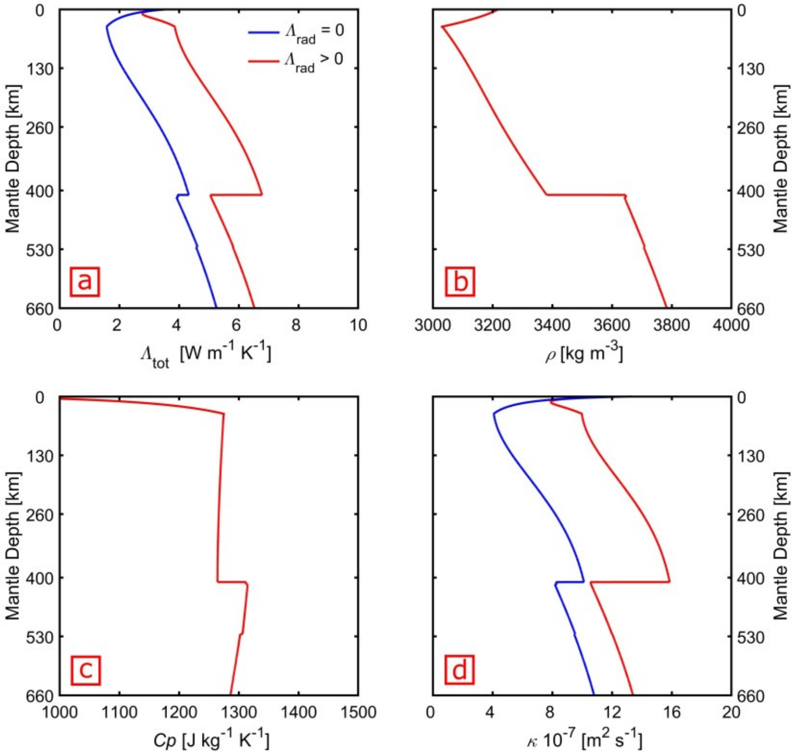

Fig. S7 | Radial profiles (0 to 660 km of depth) of different thermodynamic parameters computed along the linear T gradient of the lithosphere (35 K/km) and the adiabatic P, T profile of the mantle[20,25]. The subplots represent: a) total thermal conductivity $\Lambda_{tot}[W\ m^{-1}\ K^{-1}]$; b) density $\rho[kg\ m^{-3}]$, computed using (S13)-(S19); c) specific heat capacity $Cp[J\ kg^{-1}K^{-1}]$, computed using (S20)-(S23); d) heat diffusivity $\kappa[m^2 s^{-1}]$, computed using eq. (S24).

Physical Model. We represented the slab as a rectangular object with a length $L_{slab} = 6 \times 10^5\ m\ (600\ km)$ and a thickness H_{slab} (Fig. S8). Slab thickness varies as a function of its age t_{slab}. Therefore, we computed H_{slab} using the analytical solution of thermal boundary layer thickness reported by Ref.[43]:

$$H_{slab} = 2.32\sqrt{\kappa t_{slab}} \tag{S25}$$

Where t_{slab} represents the slab age in seconds ($1\ Myrs = 3.154 \times 10^{13} s$), and κ is the thermal diffusivity, here set to a constant value of $\kappa = 1 \times 10^{-6}\ m^2\ s^{-1}$. The square product $\sqrt{\kappa t_{slab}}$ represents the characteristic thermal diffusion distance[43]. The maximum age of $80\ Myrs$ was chosen because, above this value, the analytical solution does not produce a slab thicknesses that match the geophysical observations[44]. We selected four different slab ages t_{slab}: [20; 40; 60; 80] $Myrs$, which approximately correspond to four slab thicknesses H_{slab}: [60; 80; 100; 120] km.

We computed the initial slab temperature profile at the trench with the half-space cooling equation[43]:

$$T_x = T_0 + erf\left(\frac{x}{2\sqrt{\kappa t_{slab}}}\right) * (T_m - T_0) \tag{S26}$$

To solve the equation, we assumed a surface temperature of $T_0 = 300\ K$, and an ambient mantle temperature of $T_m = 1600\ K$. Equation (S26) is one-dimensional, and it produces a temperature profile along the spatial coordinate x $[m]$, which represents the slab thickness. We truncated the solution of equation (S26) at H_{slab}, and we extended the 1D temperature transect to the rest of the slab, along its length L_{slab} (Fig. S8). The resulting temperature field was taken as initial conditions for each slab. The assumption of using a constant heat diffusivity value in equations (S25) and (S26), is justified by the necessity to create identical slab thicknesses H_{slab} and initial temperature T_x profiles for the two tested cases ($\Lambda_{rad} = 0$; $\Lambda_{rad} > 0$), and to limit our investigation only to the effects of olivine's radiative thermal conductivity on the thermal evolution of subducting slabs.

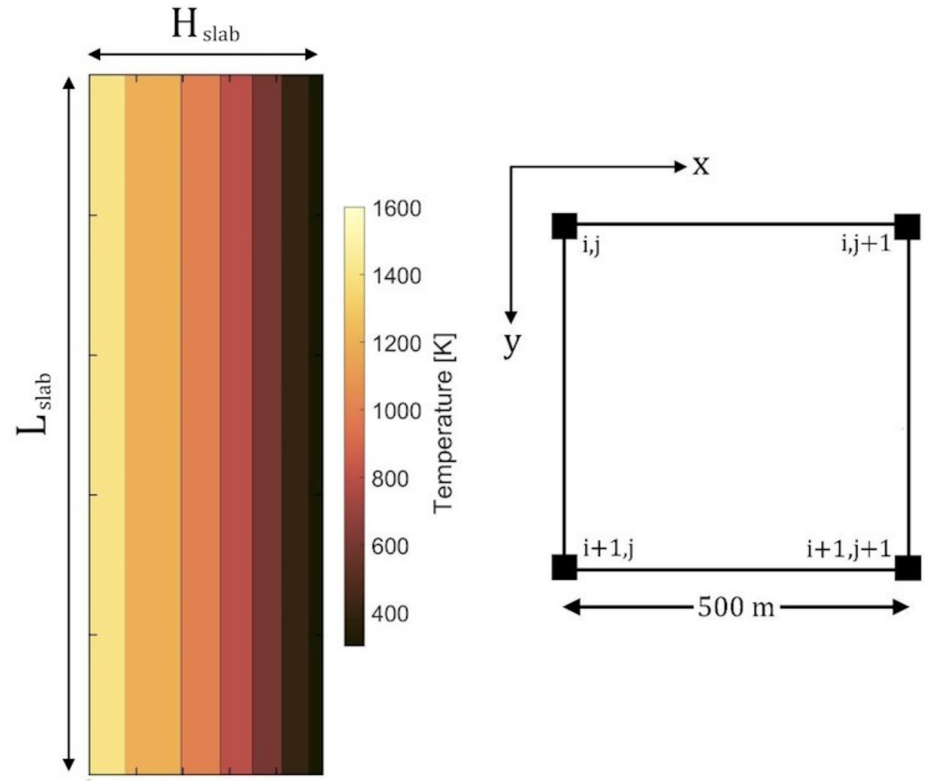

Fig. S8. | Representation of a slab in our models, and schematics of the grid cell used to discretize the slab. The slab was simplified as a $H_{slab} \times L_{slab}$ rectangle, with L_{slab} fixed at $600\ km$. We computed H_{slab} with an age-dependent analytical solution[43], see eq. (S25). The colors inside the slab illustrate the initial temperature field computed with the analytical solution for the half-space cooling[43], see eq. (S26). To plot temperature, we used the Scientific Color Maps[45], in which dim colors represent areas with low T, whereas bright colors indicate high T. We used square grid cells to discretize the slab. Each cell is bounded by 4 nodes at the corners, and the grid spacing between each node is set to a constant value of $500\ m$. The nodes are identified by the $[i, j]$ coordinate system, in which i identifies the rows (y-direction) and j identifies the columns (x-direction).

The code computes the thermal evolution of the slab from H_{slab} down to a prescribed depth D_{end} (Fig. S9). For this study, we set $D_{end} = 660\ km$, which represents the bottom of the MTZ. To limit the size of the model and minimize the computational time, we choose $L_{slab} < D_{end}$. Moreover, we set H_{slab} as the entry depth of the slab in the mantle, to avoid simulating complex interactions between the slab and the overriding plate at the trench. Slab subduction was simplified by prescribing a constant sinking velocity v_{sink} to a slab with vertical dip ($\theta_{dip} = 90°$). The sinking velocities used in this study were v_{sink}: [2.5; 5.0; 7.5; 10; 12.5] $cm\ yr^{-1}$, leading to a sinking time to reach 660 km of depth of t_{sink}: [21.6; 10.8; 7.2; 5.4; 4.3] $Myrs$.

During subduction, the heat flows from the hot mantle toward the cold inner regions of the slab. The heat diffusion is derived from the Fourier's Law[46] of heat conduction $Q = -\Lambda \nabla T$:

$$\rho Cp \left(\frac{\partial T}{\partial t} \right) = -\nabla \cdot (-\Lambda \nabla T) \tag{S27}$$

This equation describes the temperature evolution over time $(\partial T / \partial t)$ $[K\ s^{-1}]$, caused by a temperature gradient in 3D space (x, y, z) ∇T $[K\ m^{-1}]$. The amount of thermal energy that enters or leaves an object is quantified by the heat flux $Q [W\ m^{-2}]$. In diffusion processes, the heat flux is controlled by thermal diffusivity κ $[m^2 s^{-1}]$, which is computed from the thermodynamic parameters of the material as $\kappa = \Lambda / \rho Cp$: density ρ $[kg\ m^{-3}]$, specific heat capacity Cp $[J\ kg^{-1} K^{-1}]$, and thermal conductivity Λ $[W\ m^{-1} K^{-1}]$. The length covered by the propagating heat in the thermal diffusion process increases with the square root of time $L \propto \sqrt{\kappa t}$. In 2D, the heat diffusion equation becomes:

$$\rho Cp \left(\frac{\partial T}{\partial t} \right) = \frac{\partial}{\partial x} \left(\Lambda_H \frac{\partial T}{\partial x} \right) + \frac{\partial}{\partial y} \left(\Lambda_V \frac{\partial T}{\partial y} \right) \tag{S28}$$

In our model, the x-direction represents the horizontal axis (along H_{slab}), whereas the y-direction represents the vertical axis (along L_{slab}), (Fig. S8). Note that, given the geometry of the present simulation, the vertical direction coincides with the radial direction i.e., toward the center of the Earth. The code can distinguish between horizontal Λ_H and vertical Λ_V thermal conductivity thus enabling models of anisotropic thermal conductivity. In this study, however, we ignored the anisotropic behavior of olivine[47,48] and we set $\Lambda_H = \Lambda_V$.

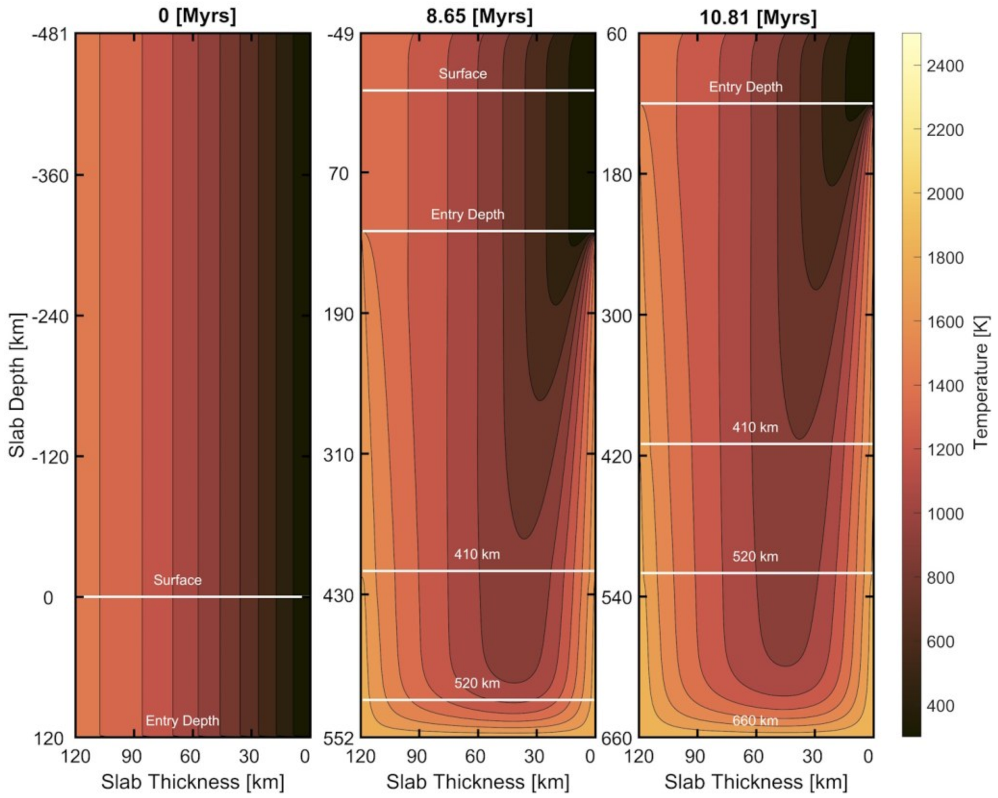

Fig. S9. | Representation of slab subduction in our models. Temperature field is plotted using the Scientific Color Maps[45]. The cold slab subducts vertically ($\theta_{dip} = 90°$) – with constant sinking velocity v_{sink} – inside the hot mantle. In the model we can prescribe a maximum depth of subduction, which we set to $660\ km$ for this study. Each subplot shows a different timeframe of slab's thermal evolution. Note that this is just a simplified slab subduction: our model is purely thermal, and we computed the mantle just as a boundary condition: i.e., as one extra stencil of nodes outside of the slab (see Fig. S11).

Numerical Model. We employed the finite difference (FD) method[49] to solve the partial differential equation for 2D heat diffusion equation (S28). To do so, we discretized the domain into square cells with a constant grid space $\Delta x = \Delta y = 500\ m$ (Fig. S8). The number of nodes along the y-direction was fixed at 1202, whereas the number of nodes along the x-direction depended on the slab thickness: [122;162;202;242] nodes. For the calculations we opted to use the central difference for its high accuracy[49]:

$$\rho_i Cp_i \left(\frac{\Delta T_i}{\Delta t}\right) = \frac{1}{\Delta x^2}\left[\Lambda_B\left(T_{i,j+1} - T_{i,j}\right) - \Lambda_A\left(T_{i,j} - T_{i,j-1}\right)\right] + \frac{1}{\Delta y^2}\left[\Lambda_\beta\left(T_{i+1,j} - T_{i,j}\right) - \Lambda_\alpha\left(T_{i,j} - T_{i-1,j}\right)\right] \quad \text{(S29)}$$

In this formulation, the temperature variations in a central node T_{ij} are computed considering the four neighboring nodes: $T_{i-1,j}$ (y-upper), $T_{i+1,j}$ (y-lower), $T_{i,j-1}$ (x-left), and $T_{i,j+1}$ (x-right) (Fig. S10).

Thermal conductivity is the most important parameter of the equation because it is a flux-controlling parameter i.e., it controls the flow of energy into the system $[W\ m^{-1}\ K^{-1} = J\ s^{-1}\ m^{-1}\ K^{-1}]$. Therefore, it must be treated carefully in order to avoid the formation of an artificial flow of energy at any sharp Λ discontinuities. To avoid this eventuality, we implemented a conservative discretization[49], which employs the average thermal conductivities between two adjacent nodes (Fig. S10): Λ_A, Λ_B represent the horizontal thermal conductivities at the intermediate positions $A\ [i, j - \frac{1}{2}]$ and $B\ [i, j + \frac{1}{2}]$, whereas Λ_α, Λ_β represent the vertical thermal conductivities at the intermediate positions $\alpha\ [i - \frac{1}{2}, j]$ and $\beta\ [i + \frac{1}{2}, j]$. On the other hand, density ρ_i and specific heat capacity Cp_i were computed at central node $[i, j]$.

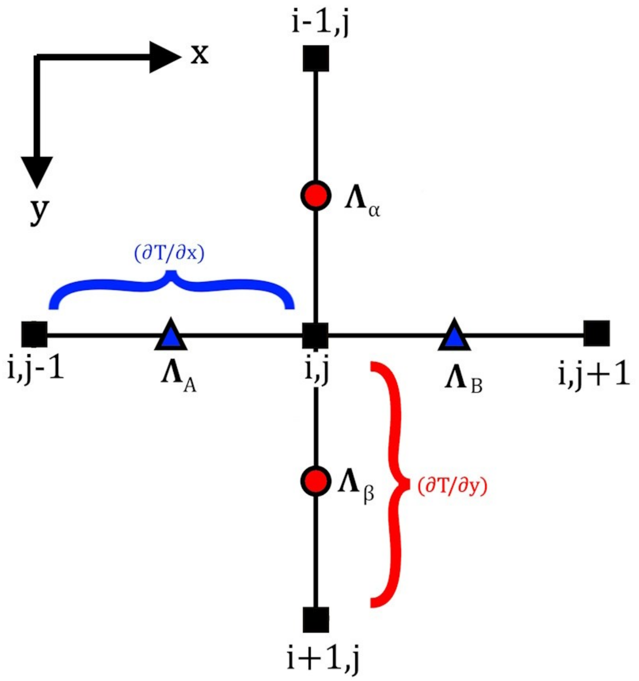

Fig. S10. | Schematics of the central difference method. The grid nodes are represented as squares with $[i, j]$ indexing. Auxiliary nodes in mid space are represented as blue triangles (x–direction), and red circles (y–direction). The thermal conductivities in A and B, are used to calculate the horizontal temperature gradient (blue curly bracket), whereas the conductivities in α and β are used to calculate the vertical temperature gradient (red curly bracket).

The time step Δt depended on the sinking velocity, and it was computed to assure that the slab would have descended of exactly $100\ m$ after each Δt. The employed time steps were Δt: ~[4000;2000;1300;1000;800] yrs. Even for the largest time step, the diffusive length scale ($L_{diff} = \sqrt{\kappa \Delta t}$) is only $355\ m$, which is less than the employed grid space. The code was benchmarked for the prescribed Δx and for each Δt.

We prescribed an extra stencil of nodes at the four sides of the slab to form the boundaries of the model. At depths greater than H_{slab} (i.e., slab entry point in the mantle) the boundaries represent a 500-m-thick layer of ambient mantle (Fig. S11). To simulate the hot mantle environment, we prescribed isothermal conditions at the boundaries of the domain. The boundary temperature is updated after each time-step, to reproduce the adiabatic temperature increase[20] of the mantle. This ΔT is also added to the slab temperature. Moreover, pressure changes at depth are taken from the PREM model[25]. At depths shallower than $120\ km$ the

boundaries were designed to maintain the slab at the initial 1D temperature profile T_x, see equation (S26).

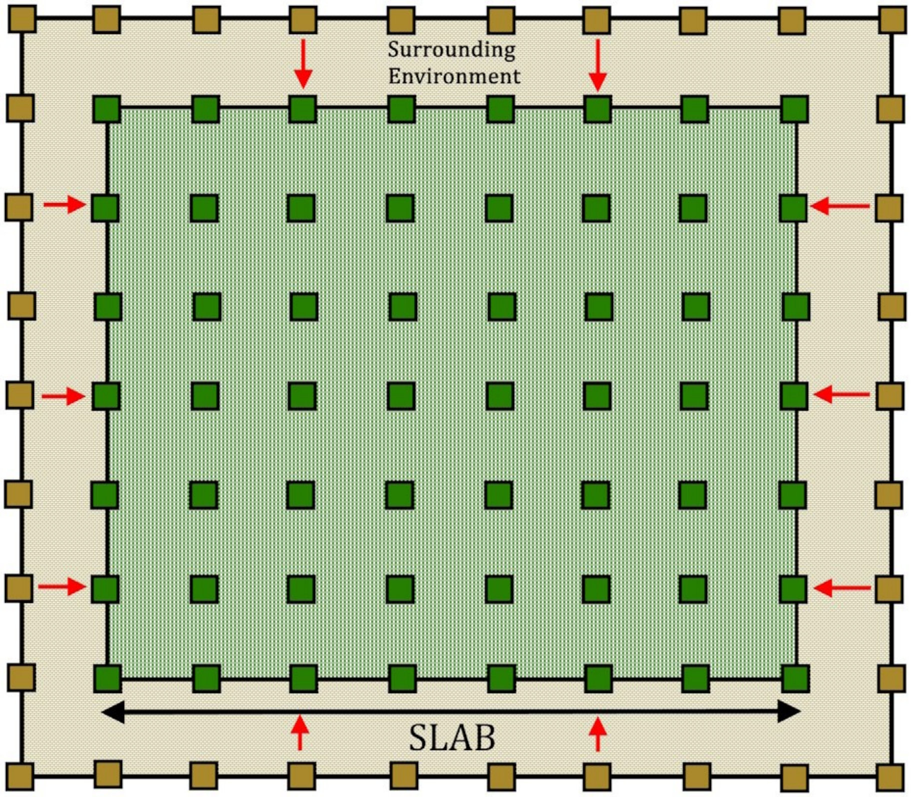

Fig. S11. | Schematics of the domain subdivision in our model. Most of the domain is represented by the slab (green area), in which we can prescribe L_{slab} length and width H_{slab}. The slab can be subdivided into different lithologies (e.g., oceanic crust, hydrous layer) with variable thickness. In our case the slab is only made by one monomineralic lithology (see main text). The surrounding environment (surface lithosphere or deep mantle) is implemented simply as an extra stencil of nodes at the four sides of the slab (light brown area). The red arrows represent the heat flux directed from the hot environment toward the cold slab.

Model strengths and limitations. Here listed are the limitations of our model design, which are also reported in Ref.[50]:

(A) Model dimensionality. Our model is limited to solve the heat diffusion equation in 2D, whereas subducting slabs are 3D objects. As the number of dimensions increases, the directions from which the heat flow is established also increase: two directions in 1D (left and right); four directions in 2D (left, right, up, and down); and six directions in 3D (left, right, up, down, front, and back). An initially cold 3D body surrounded by a hot environment is heated up faster than in the 2D case, which has less heat flow pathways. Consequently, in a 3D model, the faster heating will enhance the difference between Λ_{lat}-only and Λ_{rad}-including models, due to the non-linear T-dependency of both conductivities. However, in the case examined in this study, that is a subducted slab, we expect that the relative difference between the two sets of models will not change significantly. In our simulations, we solved the heat diffusion along the slab thickness, which is its shortest dimension and the one with the strongest temperature gradient ΔT (i.e., the most relevant dimension for diffusion problems). The width of a slab segment (3rd dimension) is typically much longer than its thickness (up to 10^3 *km*) and the temperature gradient along this dimension is significantly weaker. The main conclusion of this work, therefore, is robust.

(B) Model domain. Our model domain is limited to the subducting slab, which is bounded by a 500-*m*-thick layer of surrounding environment. This limited domain does not include two key features of a subducting zone: the overriding plate and the mantle wedge. The subducting slab is mechanically coupled to the overriding plate down to a depth of ~ 60 *km*. The complex interactions between the two plates influence the thermal evolution of the slab in the shallowest part of the subduction zone. In our model, subduction starts at 60-120 *km* of depth to avoid simulating these interactions. Moreover, limiting size of the surrounding environment forbids the formation of the characteristic "halo" of cold mantle material that surround subducting slabs. This feature is a consequence of diffusive slab heating which removes heat from the environment, thus causing local cooling of the mantle wedge. Corner-flow models of the mantle wedge[51,52], report that slab subduction induces the upwelling of hot mantle material from deeper mantle regions while dragging the cooled mantle material downward. This process creates a "cold nose" that can

potentially maintain the slab surface at relatively low temperature at $> 100\ km$ of depth[51,52]. Given the non-linearity of this physical problem, we are not able to determine whether the temperature profiles computed in this study represent a cold or a hot endmember. Future models should include the presence of the overriding plate, the changes of ambient mantle temperature as the slab heats up, and the corner flow in the mantle wedge.

(C) Subduction geometry. For more realistic dip angles ($20° < \theta < 60°$), the path travelled by the slab to reach the terminal depth D_{end} would be longer: $D_{\theta} = D_{end}/sin\theta$, than in our vertically subducting model ($\theta = 90°$; $D_{\theta} = D_{end}$). Consequently, at D_{end}, a slab subducting at an angle $< 90°$ would be warmer compared to our model.

(D) Simplified physics. Our code is designed to uniquely solve heat diffusion problems; hence our subduction model is purely thermal. Comprehensive slab subduction models should also compute the density-dependent gravitational pull (variable sinking velocity) and viscoplastic rheology (temperature- and stress- dependent viscosity).

(E) Simplified petrology. In our model we used a simplified petrology, by assuming the slab to be exclusively composed by olivine and its high-P polymorphic modifications: wadsleyite and ringwoodite. The aggregate thermal conductivity of slab lithologies should be calculated including the contribution of other major phases: clino- and ortho-pyroxenes (cpx, opx), garnets (grt); Al-rich phases (e.g., phlogopite, phengite, chlorite), SiO_2-polymorphs (coesite, stishovite), and hydrous phases (e.g., antigorite talc, brucite, humite group). Unfortunately, for most of these minerals, thermal conductivity measurements at the relevant P, T conditions of the mantle are not available. The main goal of this study is to provide reliable estimates of radiative thermal conductivity for olivine, the most relevant mineral in the upper mantle and in subducting lithosphere. Our measurements, however, give an upper boundary estimate of the aggregate radiative thermal conductivity of upper mantle rocks, because the presence of opaque minerals or regions with extremely fine grain will significantly reduce Λ_{rad}^{UM}. Nonetheless, the radiative thermal conductivities of orthopyroxenes Λ_{rad}^{opx}, clinopyroxenes Λ_{rad}^{cpx}, and garnets Λ_{rad}^{grt} reported by Ref.[35] are expected to be similar to olivine's at $T > 1500\ K$. The estimated aggregate radiative conductivity of pyrolite Λ_{rad}^{pyr} (ol, opx, cpx, grt) and meta-basalts Λ_{rad}^{MORB} (opx, cpx, grt) with a grain size of 0.001-1 cm, at the P, T conditions of the upper mantle, is ranging

between $1 < \Lambda_{rad} < 3.5\ W\,m^{-1}\,K^{-1}$ (Ref.[8]). It should be noted, however, that these estimates are based on experimental data that do not consider all relevant light absorption mechanisms at the high-T, hence they should be taken with caution. Moreover, the aggregate thermal conductivity of a rock should be computed considering phase abundances in each lithology at the given P-T conditions.

(F) Absence of additional heat sources. Future model should also include the contribution of other heating sources to slab heating, e.g.: radioactive heat production, frictional heating, and positive/negative latent heat generated from exothermic/endothermic phase reactions.

Despite the assumptions and simplifications, our model effectively captures the basic physics underlying non-linear heat diffusion in the slab. To further investigate the large-scale effects of Λ_{rad} on slab thermal evolution, radiative heat transport must be incorporated into fully coupled thermo-mechanical models.

Code benchmark. The numerical solution of the heat diffusion was benchmarked against the analytical solution of a 2D Gaussian temperature distribution problem (see the Supplementary Material of Ref.[50] for more details):

$$T(r,t) = \frac{T_{max}}{\sqrt{1+\frac{4\kappa t}{\sigma^2}}} exp\left(\frac{-r^2}{\sigma^2+4\kappa t}\right) \quad \text{(S30)}$$

$$r = \sqrt{x^2+y^2}$$

where $T(r,T)$ is the position- and time-dependent temperature, T_{max} and σ are the peak temperature and the amplitude of the Gaussian profile, κ is the thermal diffusivity. The L^2 norm was computed from the misfit M between the analytical T_a and numerical solution T_n:

$$M = T_a - T_n \quad \text{(S31)}$$

$$L^2 = \frac{\sqrt{\sum(M * \Delta x * \Delta y)^2}}{X * Y}$$

where X, Y represent the length of the domain in the two directions, and $\Delta x, \Delta y$ the respective grid spacing. As reported in Ref.[50], $L^2 << 1$, which indicates that the chosen spatial discretization is sufficient to properly solve the heat diffusion equation.

Slab Viscosity. We computed slab viscosity η using a power-law relation (Arrhenius law)[53]:

$$\eta(P,T) = \eta_0 exp\left[\left(\frac{E+PV}{RT}\right) - \left(\frac{E}{RT_0}\right)\right] \quad \text{(S32)}$$

The parameter η_0 represents the reference viscosity at the reference temperature T_0 (here set at $1600\ K$, E and V are the phase-dependent activation energy and volume for creep diffusion, P is the hydrostatic pressure, R is the perfect gas constant, and T is the target temperature. To simplify the calculation, we assumed a phase-independent activation energy and volume by using the same values for the whole slab: $E = 3.0 \times 10^5 [J\ mol^{-1}]$ and $V = 5.0 \times 10^{-6} [m^3\ mol^{-1}]$. These values represent the activation energy and volume of olivine, which are derived from the mineral physics dataset of Ref.[54]. Full summary of the physical parameters used in viscosity calculations are reported in Extended Data Tab. 5.

Slab Dehydration. We wrote a MATLAB script to track the dehydration reactions encountered by a water-bearing rock along the P-T-path produced in our models. First, we defined the composition of the rock, which we assumed to be a hydrous harzburgite in the MgO-SiO_2-H_2O (MSH) system[26]. The MSH system includes 24 phases, divided into 7 groups (Extended Data Tab. 6), and 85 phase reactions divided into 4 types:

$$\text{Hydration reactions: } A + H_2O \rightarrow B_{H_2O} \quad \text{(S33)}$$

$$\text{Dehydration reactions: } A_{H_2O} \rightarrow B + H_2O$$

$$\text{Water-passing reactions: } A_{H_2O} \rightarrow B_{H_2O}$$

$$\text{Dry reactions: } A \rightarrow B$$

The subscript H_2O indicates the water-bearing minerals. The reactions reported by Ref.[26] are expressed with the left term representing the low temperature side of equilibrium. To simplify the calculations, we avoided to include brucite, talc and the minerals of the humite group.

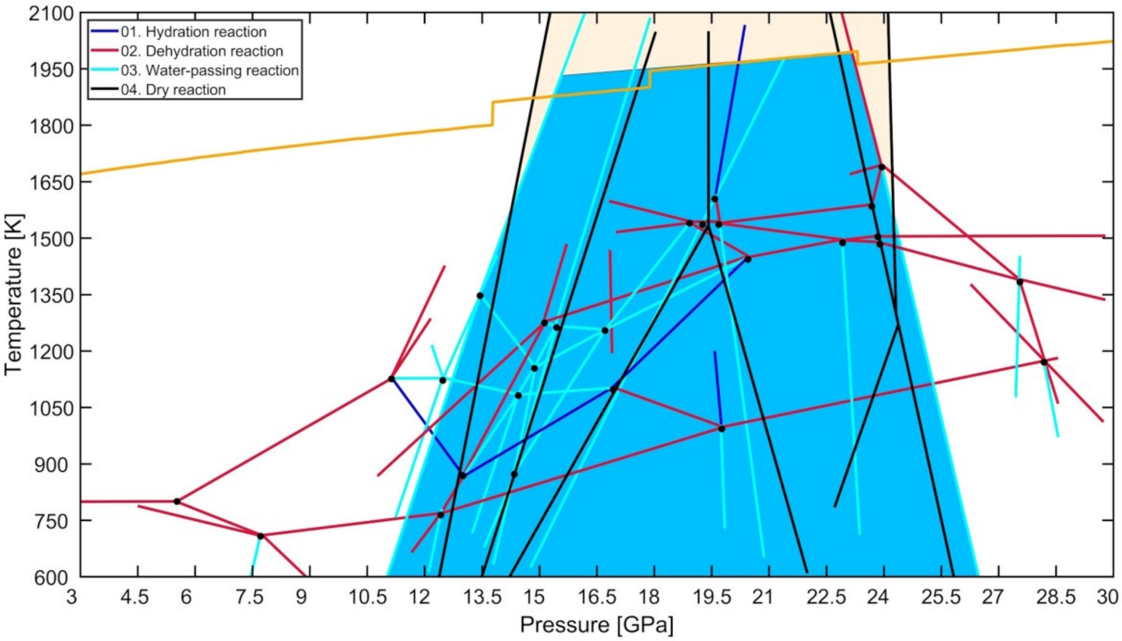

Fig. S12 | Petrogenetic grid describing the 85 univariant reactions (blue, red, cyan and black solid lines) and the 27 invariant points (black dots) of the MgO-SiO_2-H_2O (MSH) system reported by Ref.[26]. The light blue area represents the hydrous Mantle Transition Zone (MTZ), which is bounded by the (wet)wadsleyite-in reactions at low pressure, and (wet)ringwoodite-out reactions at high pressure. The sand color area represents the dry MTZ, which is bounded by the forsterite → (dry)wadsleyite reaction at low P, and the (dry)ringwoodite → bridgmanite + periclase reaction at high P. The orange line represents the mantle adiabat taken from Ref.[20].

At pressures and temperatures of the oceanic lithosphere ideal dry harzburgite in the MSH system is constituted of 80 $wt\%$ forsterite Mg_2SiO_4, and 20 $wt\%$ enstatite $MgSiO_3$[55]. The lithologies of the ocean floor are hydrated by the serpentinization reaction[26]:

$$14(Mg_2SiO_4) + 20(MgSiO_3) + 31(H_2O\ ) = 1(Mg_{48}Si_{34}O_{85}(OH)_{62}) \qquad \text{(S34)}$$

The serpentine subgroup includes the most important water bearing phases inside the slab, and antigorite $Mg_{48}Si_{34}O_{85}(OH)_{62}$ is the high-pressure polymorph of serpentine which is stable in the upper most mantle ($< 250\ km$ of depth). To compute the initial phase assemblage of the hydrous harzburgite, we assumed a 15 $wt\%$ serpentinization of the dry harzburgite, which lead to a final composition of: 68 $wt\%$ forsterite (Fo), 17 $wt\%$ enstatite (En) and 15 $wt\%$ antigorite (Atg). This level of serpentinization represents an intermediate scenario[56] where the hydrous harzburgite contains 1.85 $wt\%$ H_2O. These initial percentages correspond to an initial assemblage of Fo, En, Atg in 146: 51: 1 (mol) proportion. Antigorite composition can

be expressed by the $MgO:SiO_2:H_2O$ oxide components ratio, which is: 48:34:31. Therefore, at the initial conditions, each mol of hydrous harzburgite contains 31 mol of H_2O.

During subduction, and increasing P-T conditions, the hydrous harzburgite system undergoes a complex series of phase reactions[26] (see Fig. S13). If the P-T-path of the slab is sufficiently cold, antigorite can transform into high-pressure H_2O-bearing phases known as alphabetical phases or dense hydrous magnesium silicates (DHMS)[57]. Provided that the slab remains cold enough, the DHMS are considered the major candidate to deliver water down to the MTZ, and even deeper into the lower mantle[57]. The shallowest DHMS is PhaseA[58]: $Mg_7Si_2O_8(OH)_6$. The three most important reactions in the MSH system, branch out from the invariant point located at $P = 5.5\ GPa$ and $T = 800\ K$[26]. Depending on the P-T path of the slab there are three main scenarios. 1) hot path: harzburgite crosses the antigorite breakdown reaction ($1Atg = 14Fo + 20En + 31H_2O$); 2) intermediate path: harzburgite crosses the antigorite phase transition reaction ($5Atg = 14PhaseA + 142En + 113H_2O$), and then it crosses the PhaseA breakdown reaction ($1PhaseA + 3En = 5Fo + 3H_2O$); 3) cold path: harzburgite crosses antigorite phase transition reaction ($5Atg = 14PhaseA + 142En + 113H_2O$) and it reaches the stability field of the next DHMS (PhaseE , Phase ShyB, or PhaseD). In scenario 1) harzburgite loses all its water before 170 km of depth; in scenario 2) harzburgite loses 70% of its initial water content (from 1.85 wt% to 0.48 wt%) between 170-230 km, and it completely dehydrates within 350 km; whereas in scenario 3) harzburgite can carry water down to 660 km, at the bottom of the MTZ[26].

To compute the P-T-path of the hydrous harzburgite we took the final P-T profiles of the two sets of models ($\Lambda_{rad} = 0$ vs $\Lambda_{rad} > 0$) at two different depths inside the slab: 10 km from slab surface (corresponding to the Moho), and 30 km from the surface (corresponding to the maximum hydration depth[59] and the lower plane of the double hydrated zone)[56] (see Fig. S15). We approximated the pressure and temperature at which each reaction of the MSH system takes place with a line equation, and we limited the domain of each line within a prescribed pressure range.

$$T_{rea} = m_{rea}P_{rea} + T_0 \tag{S35}$$

$$P_1 < P_{rea} < P_2$$

The parameter represents P_{rea} the pressure (x-axis), while T_{rea} indicates the temperature (y-axis) of the reaction line. The values of m_{rea}, T_0, P, P_2, were extracted from Ref.[26] with

WebPlotDigitizer (https://apps.automeris.io/wpd/). For each recorded value of the slab P-path (P_{slab}), we computed the corresponding T_{rea} of the reaction and we compared it with the values of the slab T-path (T_{slab}). A reaction is considered crossed when $|T_{rea} - T_{slab}| < \Delta T_{max}$, where ΔT_{max} represent a maximum threshold set to $15\ K$. From the list of crossed reactions, we need to determine: a) the direction of crossing, hence the direction of the chemical reaction to distinguish the reagents and the products; b) if a reaction has effectively been crossed, depending on the presence of all the reagents among the mineral phases of the rock assembly. To determine the direction of crossing we compared the slope of the reaction m_{rea} $[MPa/K]$ with the slope of the P-T-path $m_{slab} = \Delta T/\Delta P$: if $m_{rea} < m_{slab}$ the P-T-path of the slab crosses the reaction from the low temperature side of the equilibrium (i.e. $A_{H_2O} \rightarrow B_{H_2O}$ as reported in literature); whereas if $m_{rea} > m_{slab}$ the P-T-path of the slab crosses the reaction from the high temperature side of the equilibrium (i.e. the reaction is reversed $B_{H_2O} \rightarrow A_{H_2O}$). Once the direction of the chemical reaction is determined, the script checks that all the reagents are present in the mineral assemblage. If one reagent is missing in the rock, the reaction does not occur and is removed from the list. When all the reagents are present, instead, the script finds the limiting reagent of the reaction and it normalizes the stoichiometry of the reaction accordingly. Finally, the reagents are subtracted from the mineral assemblage whereas the products are added. The amount of H_2O released by any reaction is always removed from the H_2O budget of the rock. For example:

$$\text{Initial mineral assemblage: } [146\ Fo;\ 51En;\ 1\ Atg\ (\text{with } 31H_2O)] \tag{S36}$$

$$\text{Crossed reaction: } 5\text{Atg} = 14\text{PhaseA} + 142\text{En} + 113\text{H}_2\text{O}$$

$$\text{Limiting reagent: } 1\ \text{Atg}$$

$$\text{Normalizing factor: } 1/5$$

$$\text{Normalized reaction: } 1\text{Atg} = 3\text{PhaseA} + 28\text{En} + 22\text{H}_2\text{O}$$

$$\text{Final mineral assemblage: } [146\ \text{Fo};\ 79\text{En};\ 3\text{PhaseA}\ (\text{with } 9\text{H}_2\text{O})]$$

The conservation of mass of the reaction is not strictly respected, because the stoichiometry of the normalized reaction is always rounded to the closest integer, and because the chemical formula of some minerals contains atoms in decimal units (Extended Data Tab. 6). The final list of reactions for each model is reported in the excel files attached to this paper.

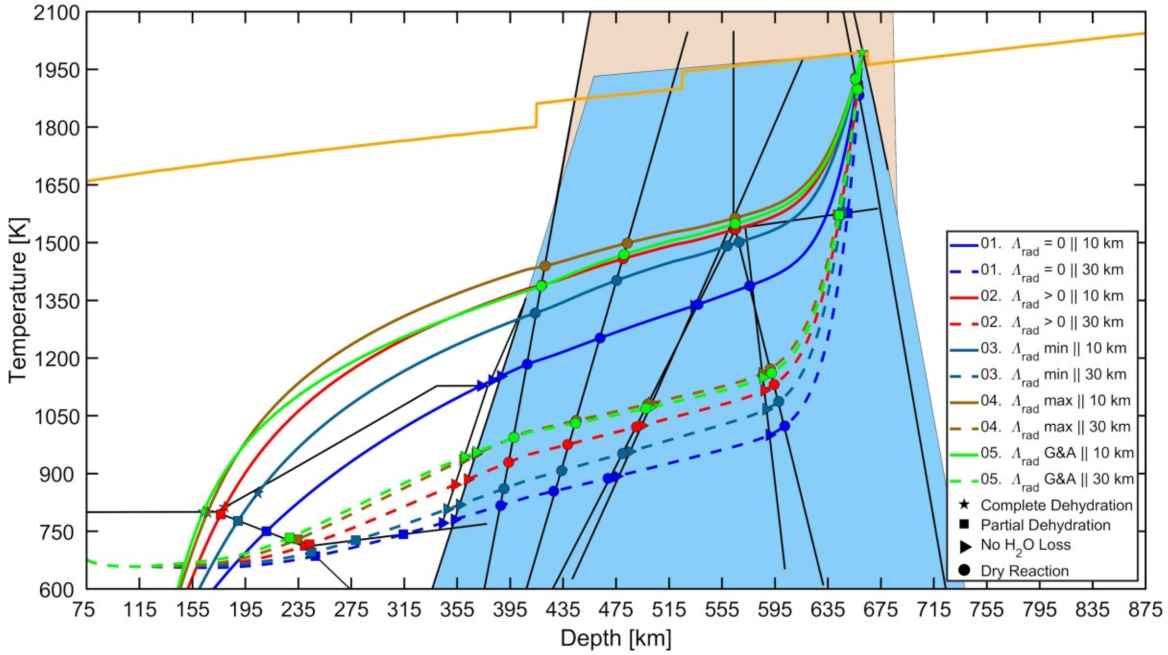

Fig. S13. | Example of slab dehydration model output computed from the slab simulation with $v_{sink} = 5\ cm\ yr^{-1}$ and $t_{slab} = 80\ Myrs$ (see Fig. 3.b in the main manuscript). This dehydration model has been computed along the P-T-path taken at 10 km (solid) and at 30 km (dashed) from the slab surface. The four colors represent the tested cases: 1) $\Lambda_{rad} = 0$ (blue); 2) $\Lambda_{rad} > 0$ (red); 3) Λ_{min} (teal) i.e., lower uncertainty bound of our Λ_{tot} estimates (i.e. -30% Λ_{rad}, -15% Λ_{lat}); 4) Λ_{max} (brown) i.e., upper uncertainty bound of our Λ_{tot} estimates (i.e. +30% Λ_{rad}, +15% Λ_{lat}); and 5) $\Lambda_{G\&A2019}$ (green), i.e. based on *Grose & Afonso, 2019,* Ref.[8] formulation computed with eq. (S12). The light blue and the sand-colored areas represent the hydrous and the dry MTZ, respectively (see caption in Fig. S12). The orange line represents the mantle adiabat[20]. Note that here, unlike Fig. S12, here we show only the phase reactions effectively crossed by the different P-T-paths. Moreover, the different type reactions reported here are not distinguished by different colors but simply plotted as black solid lines. However, the exact P, T at which a reaction is crossed and its type are indicated by different markers: (a) complete dehydration reactions (stars) i.e., the entire H_2O budget of the slab is lost; (b) partial dehydration reactions (squares) i.e., the solid reaction products are not all anhydrous and some H_2O is still retained inside the slab; (c) water-passing reaction (triangles) i.e., the entire H_2O budget of the slab is transferred from the reagents to the products and new hydrous phases are formed; (d) dry reaction (circles) i.e., H_2O-bearing phases are not involved in the reaction.

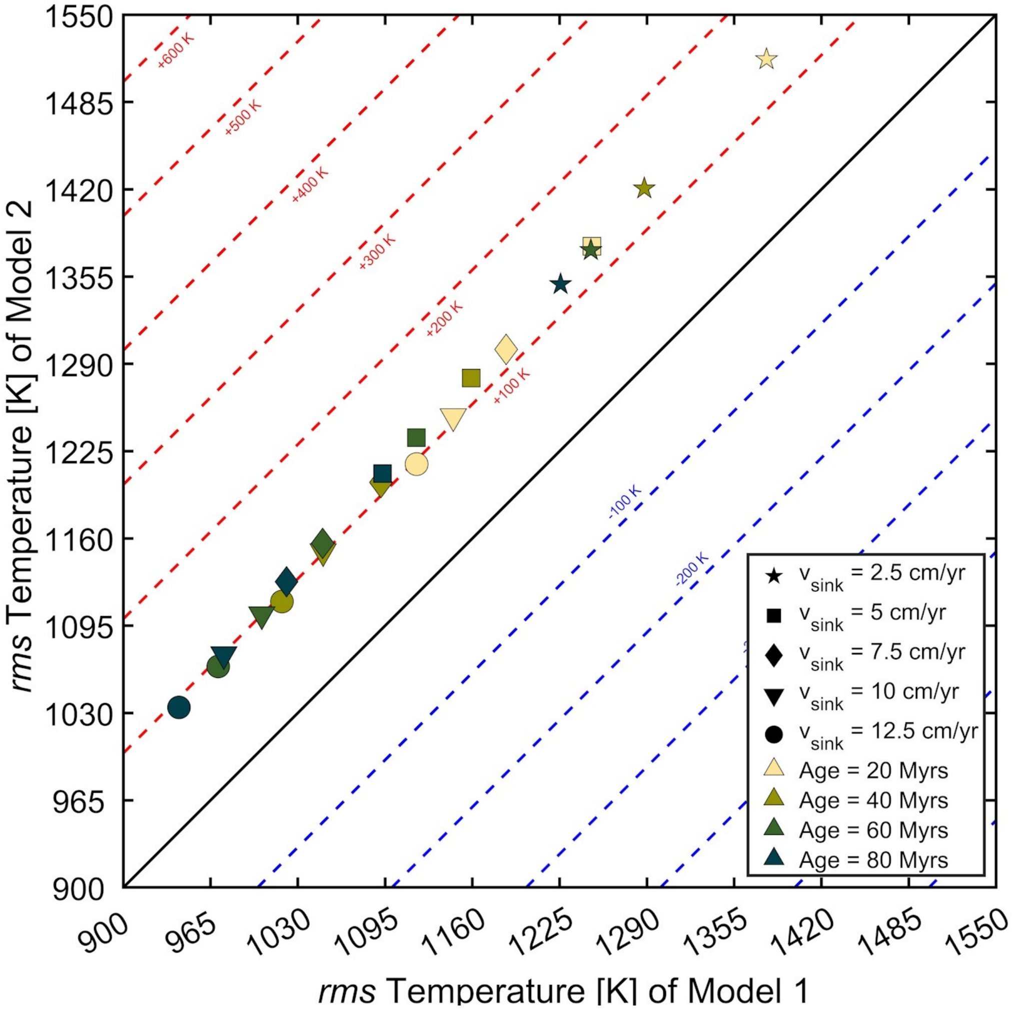

Fig. S14 | Comparison of the root-mean-square (*rms*) temperature T_{rms} $[K]$ between model set 1 ($\Lambda_{rad} = 0$) in the x-axis, and model set 2 ($\Lambda_{rad} > 0$) in the y-axis. The T_{rms} temperature is referred to the upper $40\ km$ of the slab (see main text). Each marker represents one slab characterized by a sinking velocity v_{sink} (marker shape), and a slab age t_{age} (marker color). The solid black line represents the $1:1$ correlation between the two model sets, where $T_{rms}^1 = T_{rms}^2$. We computed the temperature difference between the models as: $\Delta T_{rms} = T_{rms}^2 - T_{rms}^1$. The dashed red lines on the upper left side are hot isolines where the temperature difference is $\Delta T_{rms} > 0$. The dashed blue lines on the lower right side are cold isolines where the temperature difference is $\Delta T_{rms} < 0$. All data are reported in the Extended Data Tab. 6.

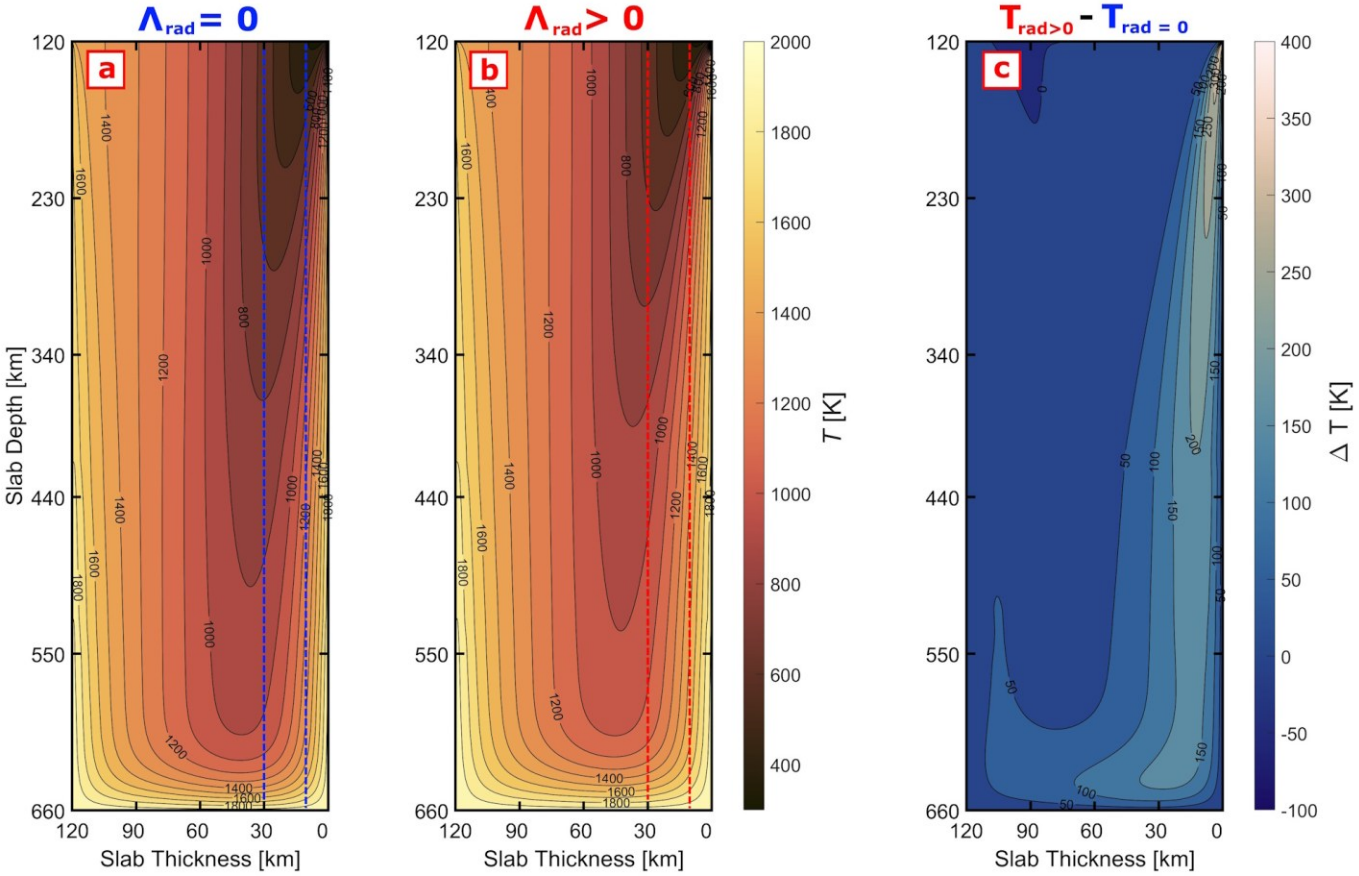

Fig. S15 | Examples of final 2D temperature distributions from a slab model with $v_{sink} = 5\ cm\ yr^{-1}$ and $t_{slab} = 80\ Myrs$ without (a) and with radiative heat transport (b). Subplot (c) shows the temperature difference between (b) and (a). Each subplot plot shows the whole thickness of the slab, starting from its surface at 0 km. For the plot we used the Scientific Color Maps[45]. The dashed lines in subplot (a) and (b) represent the final P-T profiles extracted to compute slab dehydration. These profiles were taken at: 10 km from the slab surface (corresponding approximately to the Moho), and 30 km from the slab surface (corresponding to maximum hydration depth[59]).

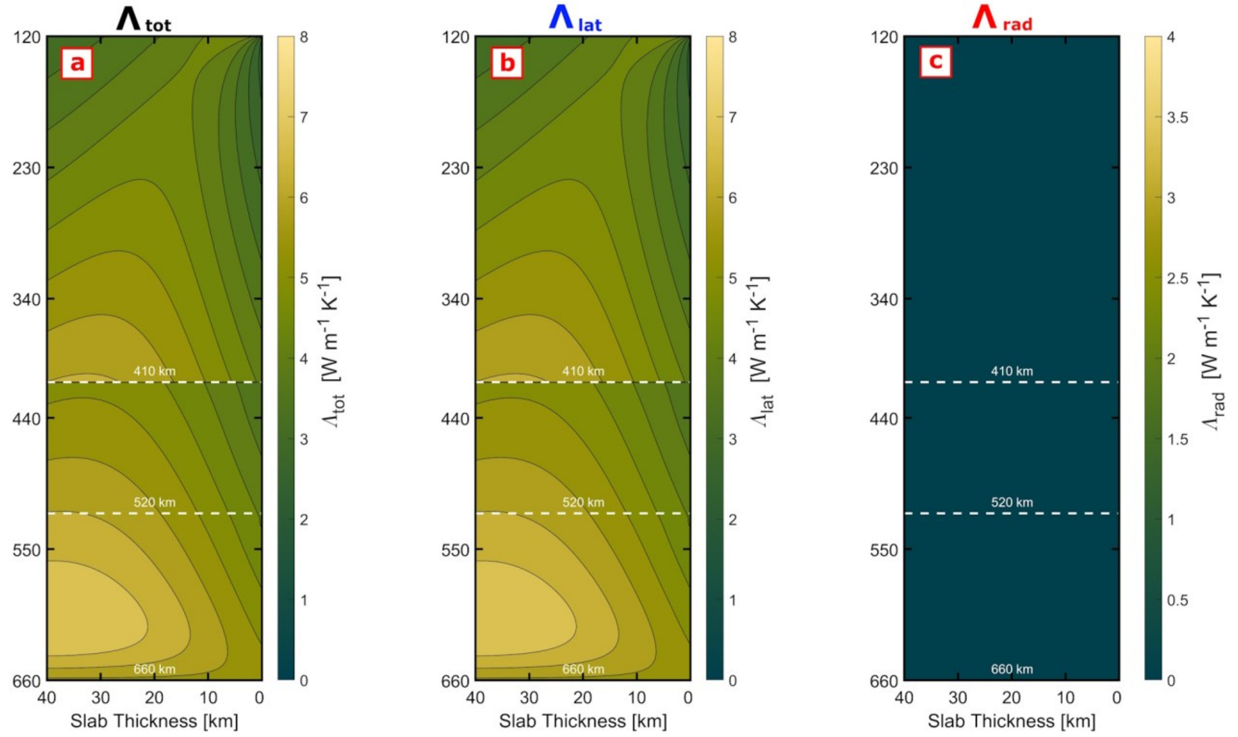

Fig. S16 | Example of contour 2D thermal conductivity fields from a slab model with $v_{sink} = 5\ cm\ yr^{-1}$ and $t_{slab} = 80\ Myrs$. In this model $\Lambda_{rad} = 0$. Each subplot shows only the upper 40 km of the slab, starting from its surface at 0 km. For the plot we used the Scientific Colour Maps[45]. Dim colours represent areas with low Λ, whereas bright colours indicate high Λ.

a. Illustrates the total thermal conductivity $\Lambda_{tot}\ [W\ m^{-1}\ K^{-1}]$ computed as $\Lambda = \Lambda_{lat} + \Lambda_{rad}$

b. Illustrates the lattice thermal conductivity $\Lambda_{lat}\ [W\ m^{-1}\ K^{-1}]$

c. Illustrates the radiative thermal conductivity $\Lambda_{rad}\ [W\ m^{-1}\ K^{-1}]$

In this case the Λ_{tot} variations across the slab thickness follows the internal temperature gradient of the slab: Λ_{tot} increases toward the inner cold core of the slab.

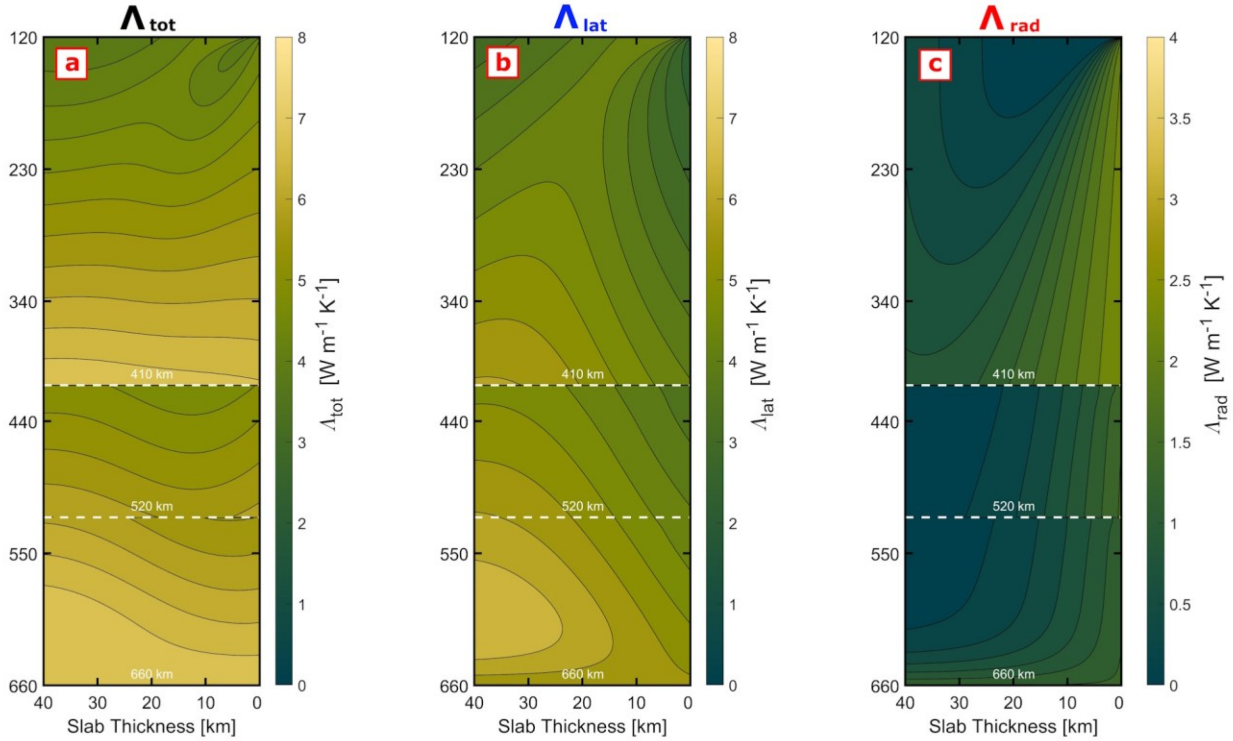

Fig. S17 | Example of contour 2D thermal conductivity fields from a slab model with $v_{sink} = 5\ cm\ yr^{-1}$ and $t_{slab} = 80\ Myrs$. In this model $\Lambda_{rad} > 0$. Each subplot shows only the upper 40 km of the slab, starting from its surface at 0 km. For the plot we used the Scientific Colour Maps[45]. Dim colours represent areas with low Λ, whereas bright colours indicate high Λ.

a. Illustrates the total thermal conductivity $\Lambda\ [W\ m^{-1}\ K^{-1}]$ computed as $\Lambda = \Lambda_{lat} + \Lambda_{rad}$

b. Illustrates the lattice thermal conductivity $\Lambda_{lat}\ [W\ m^{-1}\ K^{-1}]$

c. Illustrates the radiative thermal conductivity $\Lambda_{rad}\ [W\ m^{-1}\ K^{-1}]$

In this case the contrasting effect that temperature has on Λ_{lat} and Λ_{rad} smoothens the lateral variation of Λ_{tot}. Therefore Λ_{tot} is approximately constant across the slab thickness, whereas it steadily increases (for each Mg_2SiO_4 polymorph) with pressure.

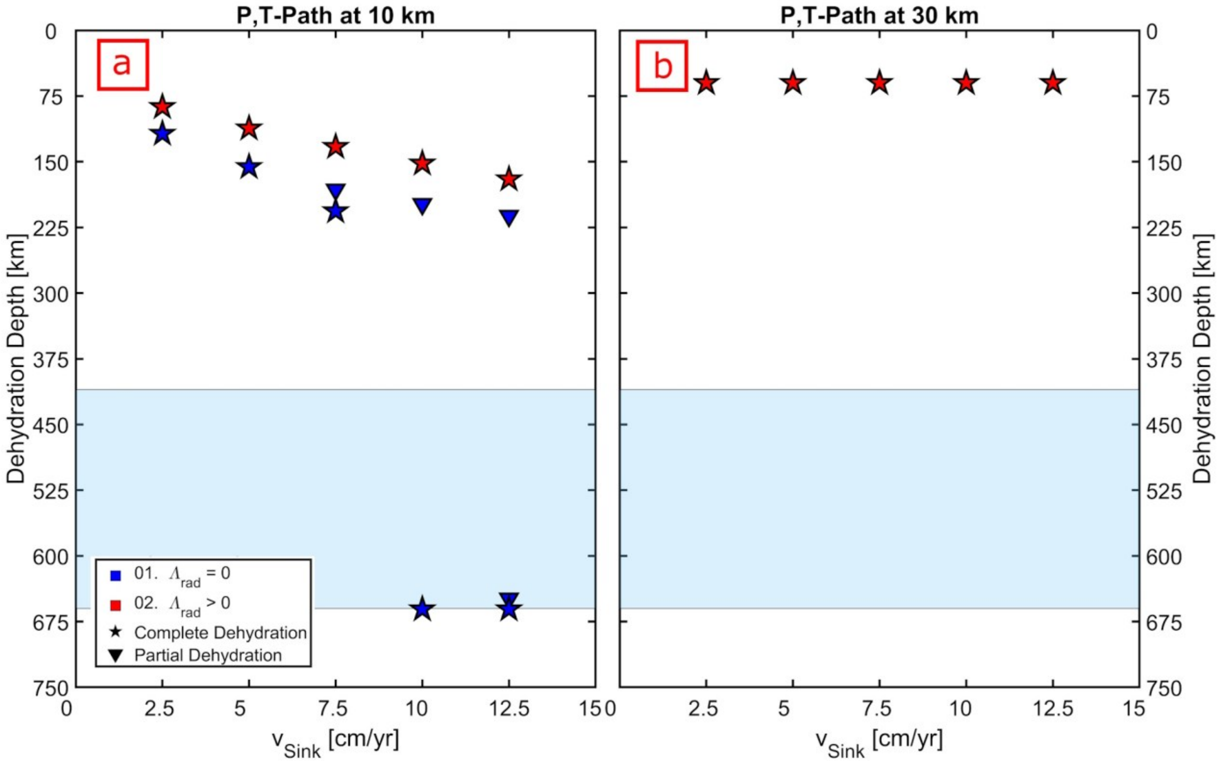

Fig. S18 | Depth of dehydration reactions as a function of sinking velocity v_{sink}, see Extended Data Tab. 7. Slab age is $t_{slab} = 20\ Myrs$, slab thickness $H_{slab} = 60\ km$. Different markers indicate the type of dehydration reaction (complete dehydration: star; partial dehydration: triangle), whereas the colors indicate the type of model ($\Lambda_{rad} = 0$: blue; $\Lambda_{rad} > 0$: red). The light blue area indicates the MTZ (410-660 km). The two subplots indicate different positions of extracted P-Tprofiles inside the slab: (a) 10 km from slab surface (Moho); (b) 30 km from slab surface (maximum hydration depth within the slab)[59]. Note that P,T-paths at 30 km are completely dehydrated already at the trench (60 km of depth). This is due to the lower thickness of young slabs (computed with eq. (S25), Ref.[43]), hence the initial temperature at 30 km within the slab ($T_{30km} = 1092\ K$, computed with eq. (S26), Ref.[43]) is higher than the breakdown temperature of antigorite at the trench $T_{atg} \sim 800\ K$, Ref.[26]. In our calculations, the first complete dehydration reaction that can be crossed by a subducting slab is the antigorite breakdown reaction: $1Atg = 14Fo + 20En + 31H_2O$ (see reaction 1 in Ref.[26]) at $T = 2.6801 \times 10^{-1}P + 7.9493 \times 10^2$ for $0 < P < 5.5263\ GPa$. In a young slab (20 $Myrs$ old), 70-100% of H_2O is lost below 200 km of depth. Only cold ($\Lambda_{rad} = 0$) and fast subducting slabs ($v_{sink} \geq 10\ cm\ yr^{-1}$) are capable to deliver ~30% of the initial H_2O content to the MTZ.

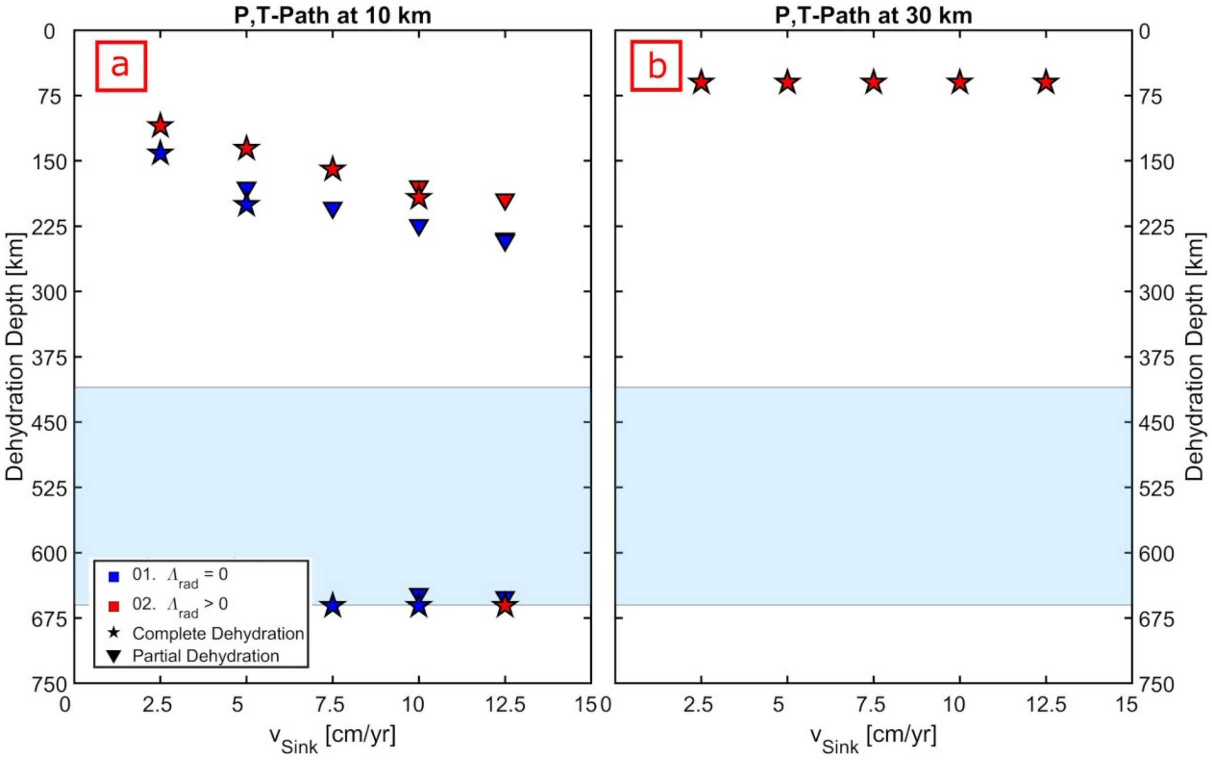

Fig. S19 | Depth of dehydration reactions as a function of sinking velocity v_{sink}, see Extended Data Tab. 7. Slab age is $t_{slab} = 40\ Myrs$, slab thickness $H_{slab} = 80\ km$. Different markers indicate the type of dehydration reaction (complete dehydration: star; partial dehydration: triangle), whereas the colors indicate the type of model ($\Lambda_{rad} = 0$: blue; $\Lambda_{rad} > 0$: red). The light blue area indicates the MTZ (410-660 km). The two subplots indicate different positions of extracted P-Tprofiles inside the slab: (a) 10 km from slab surface (Moho); (b) 30 km from slab surface (maximum hydration depth within the slab)[59]. Note that P,T-paths at 30 km are completely dehydrated already at the trench (60 km of depth). This is due to the lower thickness of young slabs (computed with eq. (S25), Ref.[43]), hence the initial temperature at 30 km within the slab ($T_{30km} = 884\ K$, computed with eq. (S26), Ref.[43]) is higher than the breakdown temperature of antigorite at the trench $T_{atg} \sim 800\ K$, Ref.[26]. In our calculations, the first complete dehydration reaction that can be crossed by a subducting slab is the antigorite breakdown reaction: $1Atg = 14Fo + 20En + 31H_2O$ (see reaction 1 in Ref.[26]) at $T = 2.6801 \times 10^{-1}P + 7.9493 \times 10^{2}$ for $0 < P < 5.5263\ GPa$. In a 40 $Myrs$ old slab, 70-100% of H_2O is lost below 200 km of depth. Cold ($\Lambda_{rad} = 0$) and fast subducting slabs ($v_{sink} \geq 7.5\ cm\ yr^{-1}$) are capable to deliver ~30% of the initial H_2O content to the MTZ. Hot slabs ($\Lambda_{rad} > 0$) can deliver ~30% of initial H_2O content to the MTZ when $v_{sink} \geq 12.5\ cm\ yr^{-1}$.

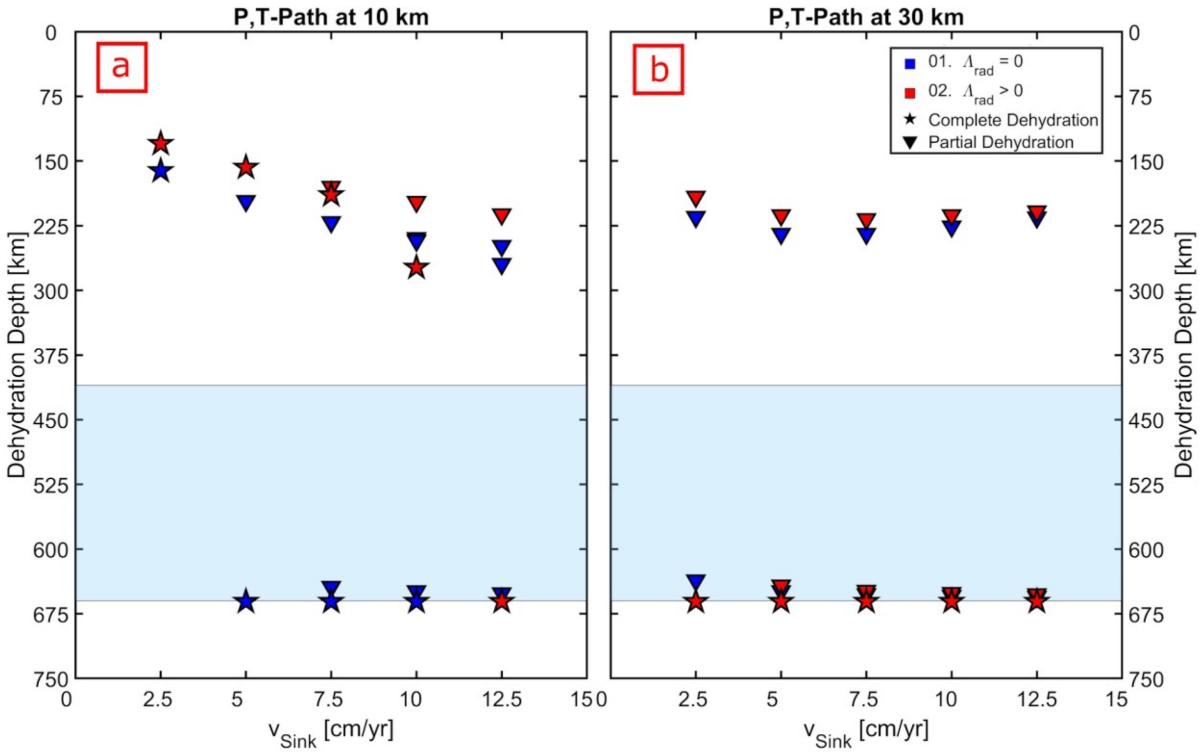

Fig. S20 | Depth of dehydration reactions as a function of sinking velocity v_{sink}, see Extended Data Tab. 7. Slab age is $t_{slab} = 60\ Myrs$, slab thickness $H_{slab} = 100\ km$. Different markers indicate the type of dehydration reaction (complete dehydration: star; partial dehydration: triangle), whereas the colors indicate the type of model ($\Lambda_{rad} = 0$: blue; $\Lambda_{rad} > 0$: red). The light blue area indicates the MTZ (410-660 km). The two subplots indicate different positions of extracted P-Tprofiles inside the slab: (a) 10 km from slab surface (Moho); (b) 30 km from slab surface (maximum hydration depth within the slab)[59]. Note that, in this case, the P,T-paths at 30 km are not completely dehydrated already at the trench (60 km of depth). This is due to the higher thickness of old slabs (computed with eq. (S25), Ref.[43]), hence the initial temperature at 30 km within the slab ($T_{30km} = 793\ K$, computed with eq. (S26), Ref.[43]) is lower than the breakdown temperature of antigorite at the trench T_{atg}~ 800 K, Ref.[26]. In our calculations, the first complete dehydration reaction that can be crossed by a subducting slab is the antigorite breakdown reaction: $1Atg\ =\ 14Fo\ +\ 20En\ +\ 31H_2O$ (see reaction 1 in Ref.[26]) at $T\ =\ 2.6801 \times 10^{-1}P + 7.9493 \times 10^{2}$ for $0\ <\ P\ <\ 5.5263\ GPa$. In a 60 $Myrs$ old slab, 70-100% of the initial water content H_2O is lost below 300 km of depth. Cold ($\Lambda_{rad} = 0$) subducting slabs ($v_{sink} \geq 5\ cm\ yr^{-1}$) are capable to deliver ~30% of H_2O to the MTZ. Hot slabs ($\Lambda_{rad} > 0$) can deliver ~30% of initial H_2O content to the MTZ when $v_{sink} \geq 12.5\ cm\ yr^{-1}$.

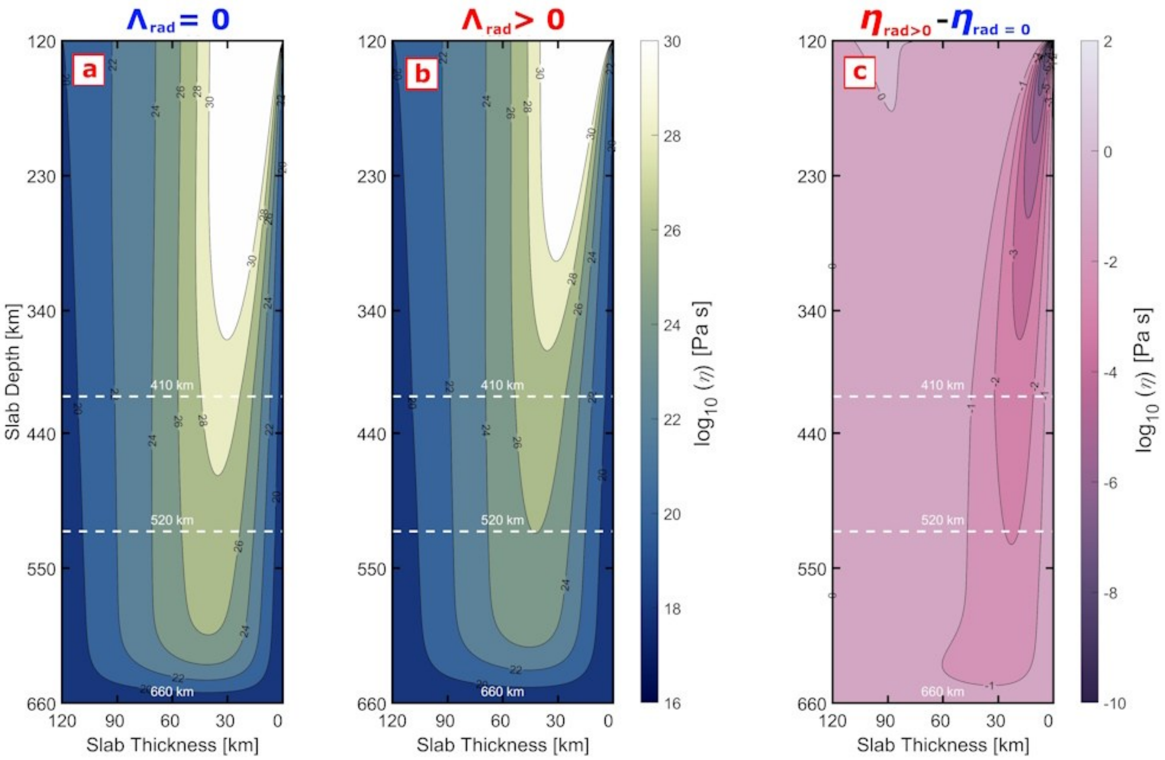

Fig. S21 | Example of contour 2D viscosity fields from a slab model with $v_{sink} = 5\ cm\ yr^{-1}$ and $t_{slab} = 80\ Myrs$. Each subplot shows the whole slab thickness ($120\ km$), starting from its surface at 0 km. For the plot we used the Scientific Color Maps[45]. Dim colors represent areas with low η and large $\Delta log_{10}(\eta)$, whereas bright colours indicate high η and small $\Delta log_{10}(\eta)$.
(a) Illustrates the log_{10} of the viscosity field $\eta\ [Pa\ s]$ of the model set 1 ($\Lambda_{rad} = 0$).
(b) Illustrates the log_{10} of the viscosity field $\eta\ [Pa\ s]$ of the model set 2 ($\Lambda_{rad} > 0$).
(c) Illustrates the difference $log_{10}(\eta_2) - log_{10}(\eta_1)$ between the two models.

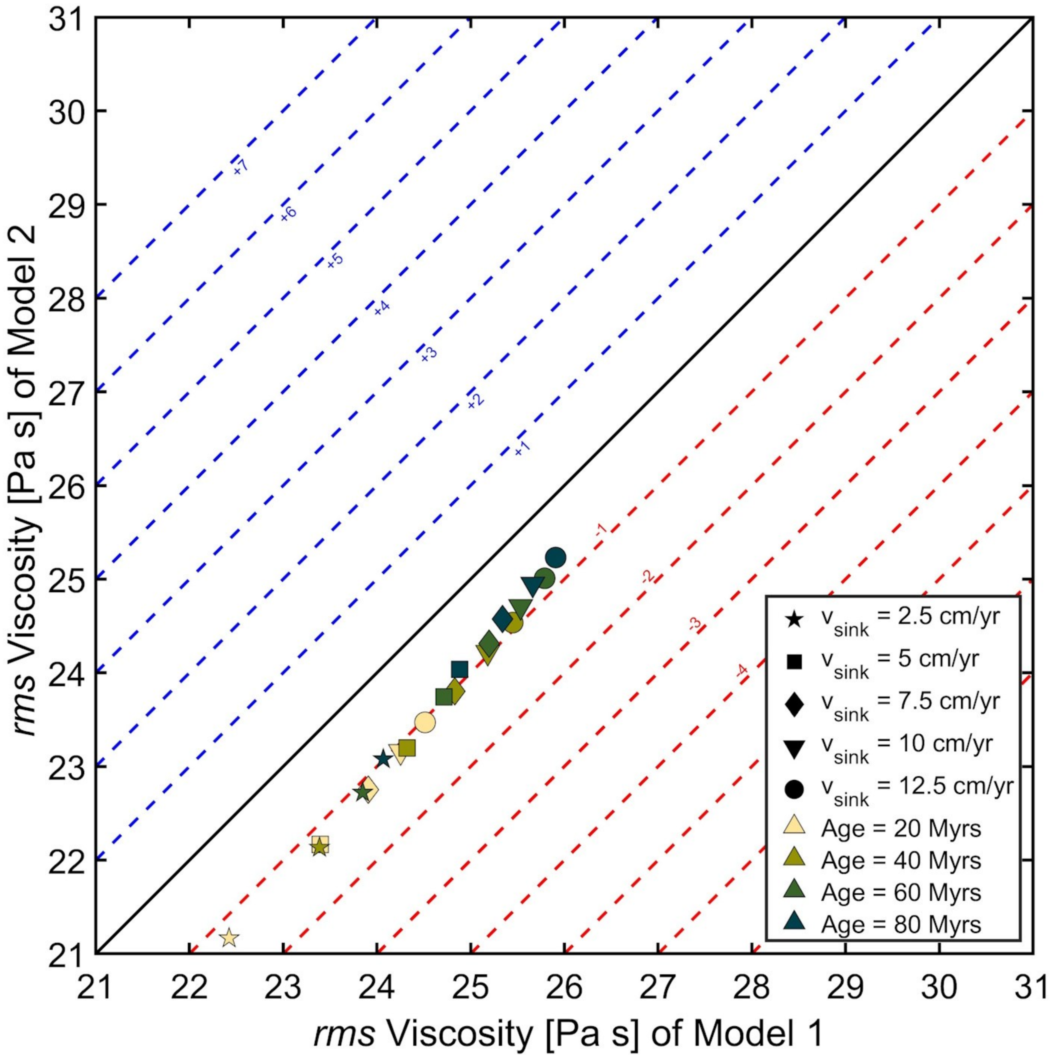

Fig. S22 | Comparison of the log_{10} of the root-mean-square (*rms*) viscosity η_{rms} $[Pa\ s^{-1}]$ between model set 1 ($\Lambda_{rad} = 0$) in the x-axis, and model set 2 ($\Lambda_{rad} > 0$) in the y-axis. The η_{rms} viscosity is referred to the upper 40 km of the slab (see main text). Each marker represents one slab characterized by a sinking velocity v_{sink} (marker shape), and a slab age t_{age} (marker color). The solid black line represents the $1:1$ correlation between the two model sets, where $\eta^1_{rms} = \eta^2_{rms}$. We computed the viscosity difference between the models as: $\Delta log_{10}(\eta) = log_{10}(\eta^2_{rms}) - log_{10}(\eta^1_{rms})$. The dashed red lines on the lower right side are the isolines where the viscosity difference is $\Delta log_{10}(\eta) < 0$ due to hotter slab temperatures. The dashed blue lines on the upper left side are the isolines where the viscosity difference is $\Delta log_{10}(\eta) > 0$ due to colder slab temperatures. All data is reported on the Extended Data Tab. 8.

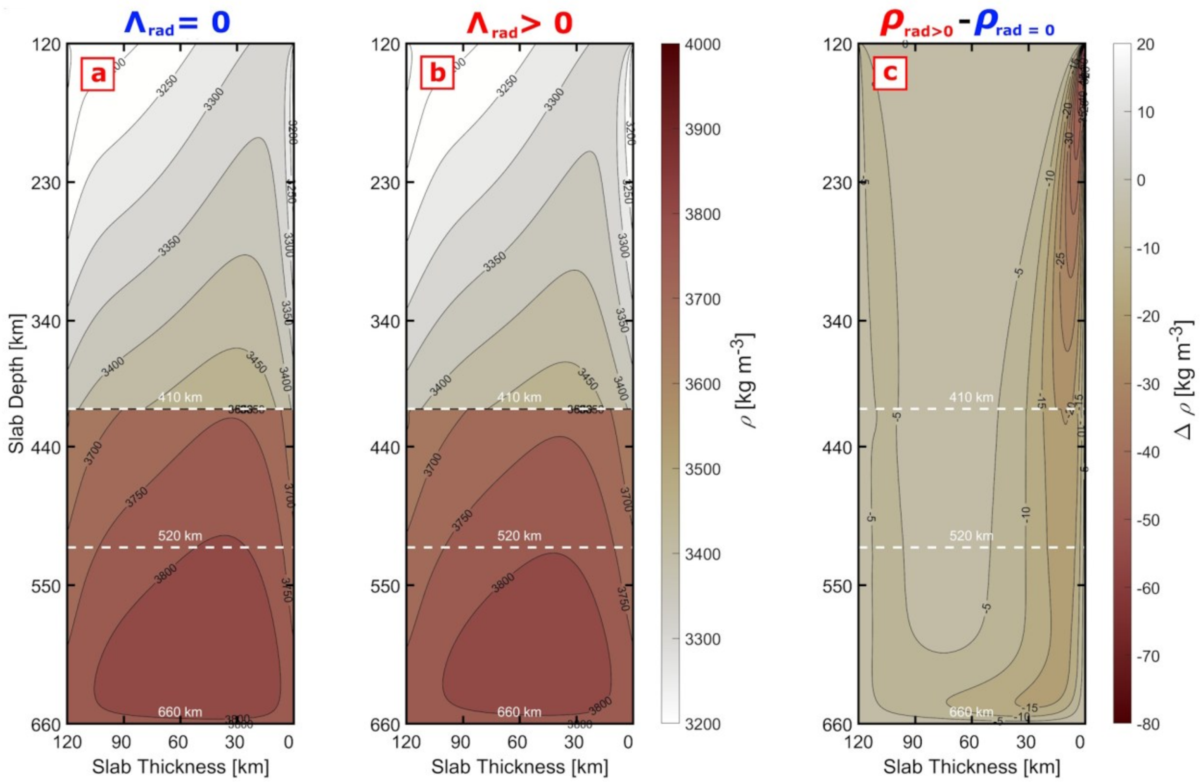

Fig. S23 | Example of contour 2D density fields from a slab model with $v_{sink} = 5\ cm\ yr^{-1}$ and $t_{slab} = 80\ Myrs$. Each subplot shows the whole slab thickness (120 km), starting from its surface at 0 km. For the plot we used the Scientific Color Maps[45]. Dim colors represent areas with high ρ and negative $\Delta\rho$, whereas bright colours indicate low ρ and positive $\Delta\rho$.
(a) Illustrates the density field $\rho\ [kg\ m^{-3}]$ of the model set 1 ($\Lambda_{rad} = 0$).
(b) Illustrates the density field $\rho\ [kg\ m^{-3}]$ of the model set 2 ($\Lambda_{rad} > 0$).
(c) Illustrates the difference $\Delta\rho\ [kg\ m^{-3}]$ between the two models.

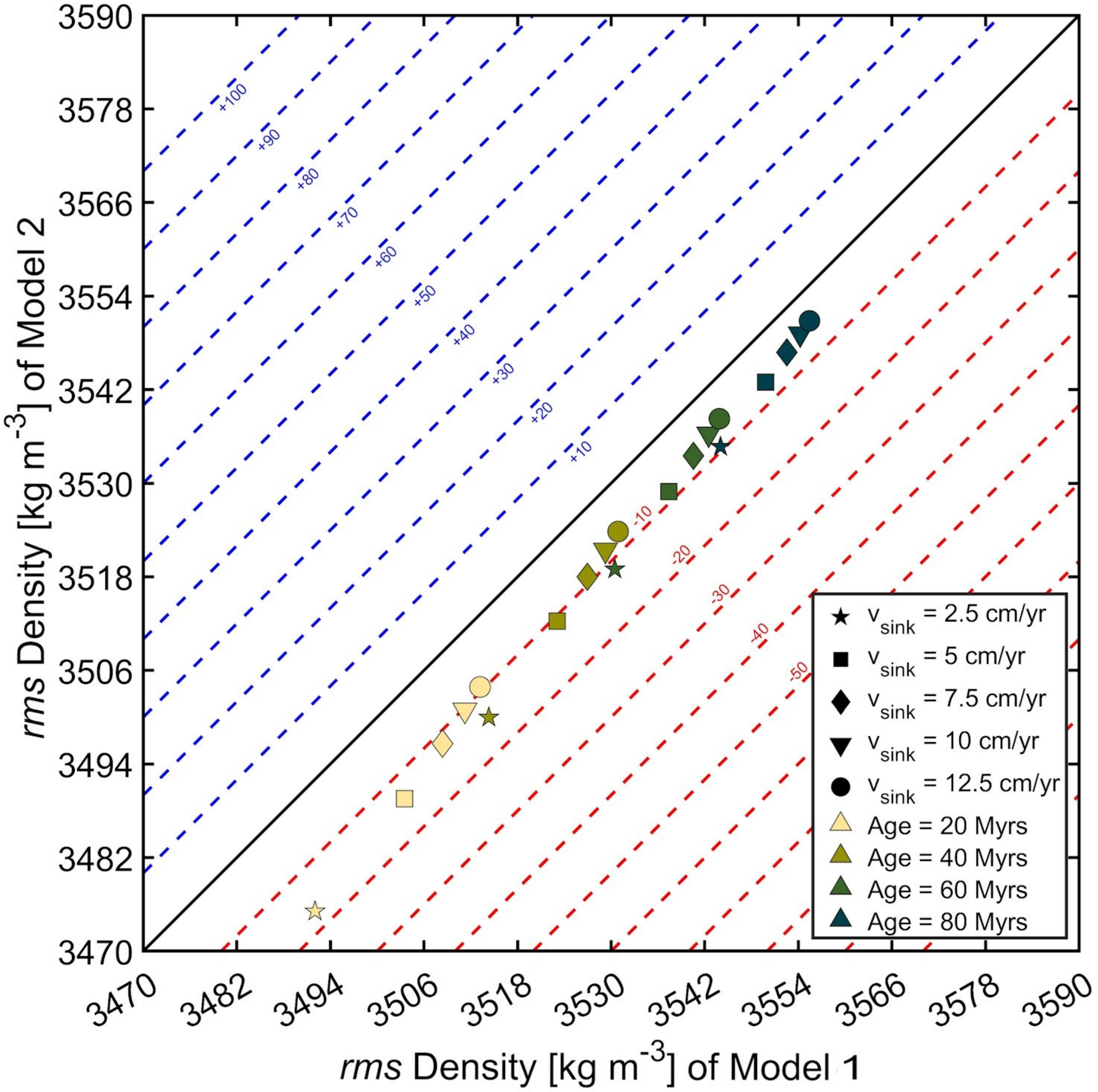

Fig. S24 | Comparison of the root-mean-square (*rms*) density ρ_{rms} $[kg\ m^{-3}]$ between model set 1 ($\Lambda_{rad} = 0$) in the x-axis, and model set 2 ($\Lambda_{rad} > 0$) in the y-axis. The ρ_{rms} density is referred to the upper 40 km of the slab (see main text). Each marker represents one slab characterized by a sinking velocity v_{sink} (marker shape), and a slab age t_{age} (marker color). The solid black line represents the $1:1$ correlation between the two model sets, where $\rho^1_{rms} = \rho^2_{rms}$. We computed the density difference between the models as: $\Delta\rho_{rms} = \rho^2_{rms} - \rho^1_{rms}$. The dashed red lines on the lower right side are the isolines where the density difference is $\Delta\rho_{rms} < 0$ due to hotter slab temperature. The dashed blue lines on the upper left side are the isolines where the density difference is $\Delta\rho_{rms} > 0$ due to colder slab temperature. All data is reported on the Extended Data Tab. 9.

633

$T\ [K]$	$\Lambda_{rad}\ [W\ m^{-1}\ K^{-1}]$		
	**~1.1 GPa**	**5 GPa**	**10 GPa**
300	0.00	0.00	0.00
400	0.02	0.02	0.02
500	0.09	0.09	0.09
600	0.21	0.21	0.21
700	0.40	0.40	0.40
800	0.62	0.62	0.62
900	0.86	0.86	0.87
1000	1.10	1.11	1.12
1100	1.33	1.34	1.35
1200	1.53	1.55	1.58
1300	1.72	1.75	1.78
1400	1.89	1.93	1.97
1500	2.05	2.09	2.13
1600	2.18	2.23	2.29
1700	2.31	2.36	2.43
1800	2.42	2.48	2.55
1900	2.53	2.59	2.67
2000	2.63	2.70	2.79
**6-th-order polynomial to compute Λ_{rad}**			
**Coefficients**	**~1.1 GPa**	**5 GPa**	**10 GPa**
$b_0\ [W\ m^{-1}\ K^{-1}]$	0.21274	0.20094	0.20094
$b_1\ [W\ m^{-1}\ K^{-2}]$	-4.8972×10^{-4}	-4.3405×10^{-4}	-4.1854×10^{-4}
$b_2\ [W\ m^{-1}\ K^{-3}]$	-3.8169×10^{-6}	-3.9335×10^{-6}	-3.9162×10^{-6}
$b_3\ [W\ m^{-1}\ K^{-4}]$	1.4051×10^{-8}	1.4105×10^{-8}	1.3946×10^{-8}
$b_4\ [W\ m^{-1}\ K^{-5}]$	-1.3445×10^{-11}	-1.3372×10^{-11}	-1.3133×10^{-11}
$b_5\ [W\ m^{-1}\ K^{-6}]$	5.3700×10^{-15}	5.3098×10^{-15}	5.1899×10^{-15}
$b_6\ [W\ m^{-1}\ K^{-7}]$	-7.8859×10^{-19}	-7.7665×10^{-19}	-7.5669×10^{-19}

Extended Data Tab. 1 | Estimated radiative thermal conductivity Λ_{rad} as a function of Temperature T. Each dataset was computed from the absorption coefficient measured at the constant pressure: ~1.1 GPa; 5 GPa; 10 GPa. In this work, radiative thermal conductivity of olivine as a function of temperature $\Lambda_{rad}(T)$ is computed using the algorithm (S6), which is bounded to a minimum and a maximum value to avoid negative or unrealistic values (Extended Data Tab. 2). Alternatively, $\Lambda_{rad}(T)$ can also be computed by employing a 6-th-order polynomial $\Lambda_{rad} = \sum_{i=0}^{6} b_i T^i$, using the coefficients $b_{1\rightarrow6}$ reported in this table.

<table>
<tr><td></td><td>Olivine</td><td>Wadsleyite</td><td>Ringwoodite</td></tr>
<tr><td></td><td>Ref.[21] - Chang et al., 2017</td><td colspan="2">Ref.[22] - Marzotto et al., 2020</td></tr>
<tr><td colspan="4">Lattice Thermal Conductivity</td></tr>
<tr><td>$\Lambda_{lat}^{bot} [W\ m^{-1}\ K^{-1}]$</td><td>3.5999999999</td><td colspan="2">4.9999999999</td></tr>
<tr><td>$\Lambda_{lat}^{max} [W\ m^{-1}\ K^{-1}]$</td><td>11.12</td><td colspan="2">16</td></tr>
<tr><td>σ_{lat}</td><td>2.4419</td><td colspan="2">3.0944</td></tr>
<tr><td>$\Lambda_{lat}^{top} [W\ m^{-1}\ K^{-1}]$</td><td>13.5619</td><td colspan="2">19.0944</td></tr>
<tr><td>y_{min}</td><td>1.280933845</td><td colspan="2">1.609437912</td></tr>
<tr><td>y_{max}</td><td>2.60726382</td><td colspan="2">2.949397662</td></tr>
<tr><td>c_0</td><td>-4.1241</td><td colspan="2">-5.4624</td></tr>
<tr><td>m_1</td><td>2.1469</td><td colspan="2">2.0791</td></tr>
<tr><td>a</td><td>-0.5</td><td colspan="2">-0.5</td></tr>
<tr><td>$\Lambda_{lat}^{n} [W\ m^{-1}\ K^{-1}]$</td><td>1</td><td colspan="2">1</td></tr>
<tr><td colspan="4">Radiative Thermal Conductivity</td></tr>
<tr><td></td><td>$This\ study - 5\ GPa$</td><td colspan="2">Ref.[19] - Thomas et al.,2012</td></tr>
<tr><td>$\Lambda_{rad}^{bot} [W\ m^{-1}\ K^{-1}]$</td><td>$10^{-10}$</td><td colspan="2">$10^{-10}$</td></tr>
<tr><td>$\Lambda_{rad}^{max} [W\ m^{-1}\ K^{-1}]$</td><td>2.70</td><td colspan="2">1.174</td></tr>
<tr><td>σ_{rad}</td><td>0.9343</td><td colspan="2">0.322</td></tr>
<tr><td>$\Lambda_{rad}^{top} [W\ m^{-1}\ K^{-1}]$</td><td>3.632</td><td colspan="2">1.496</td></tr>
<tr><td>y_{min}</td><td>-23.02585093</td><td colspan="2">-23.02585093</td></tr>
<tr><td>y_{max}</td><td>1.289885976</td><td colspan="2">0.56677082</td></tr>
<tr><td>c_0</td><td>-10.009</td><td colspan="2">-22.4290</td></tr>
<tr><td>m_1</td><td>1.8839</td><td colspan="2">3.5125</td></tr>
<tr><td>$\Lambda_{rad}^{n} [W\ m^{-1}\ K^{-1}]$</td><td>1</td><td colspan="2">1</td></tr>
</table>

Extended Data Tab. 2 | List of parameters used to compute the lattice and the radiative thermal conductivity in algorithm (S6) and (S7). Note that the coefficients for the lattice and radiative thermal conductivity of wadsleyite are assumed to be the same as ringwoodite.

Symbol	Parameter	Value
**Olivine Radiative Thermal Conductivity** – *This Study*		
σ	Stefan-Boltzmann's constant	5.670374419…×10^{-8} $W\ m^{-2}\ K^{-4}$
h	Plank constant	6.62607015×10^{-34} $J\ Hz^{-1}$
c	Speed of light	299792458 $m\ s^{-1}$
k_b	Boltzmann constant	1.380649×10^{-23} $J\ K^{-1}$
n	Refractive index	1.6
**Total Thermal Conductivity – Ref.[6]** - *Hofmeister (1999, Science)*		
Λ_0	Room temperature Λ_{lat}	4.7 $W\ m^{-1}\ K^{-1}$
a	T power-law exponent	-0.3
γ	Grüneisen parameter	1.28
α	Thermal expansion coefficient	2.75×10^{-5} K^{-1}
K_0	Bulk modulus	128.1 GPa
K_0'	Pressure derivative of Bulk modulus	4.6
f	Λ_{rad} coefficient	4.7
**Olivine Radiative Thermal Conductivity – Ref.[8]** - *Grose & Afonso (2019, G-Cubed)*		
b_0	Coefficient of Λ_{rad} $(i = 0)$	1.7821 $W\ m^{-1}\ K^{-1}$
b_1	Coefficient of Λ_{rad} $(i = 1)$	-3.1996×10^{-3} $W\ m^{-1}\ K^{-2}$
b_2	Coefficient of Λ_{rad} $(i = 2)$	-2.9458×10^{-5} $W\ m^{-1}\ K^{-3}$
b_3	Coefficient of Λ_{rad} $(i = 3)$	1.0090×10^{-7} $W\ m^{-1}\ K^{-4}$
b_4	Coefficient of Λ_{rad} $(i = 4)$	-1.0778×10^{-10} $W\ m^{-1}\ K^{-5}$
b_5	Coefficient of Λ_{rad} $(i = 5)$	4.8155×10^{-14} $W\ m^{-1}\ K^{-6}$
b_6	Coefficient of Λ_{rad} $(i = 6)$	-7.7827×10^{-18} $W\ m^{-1}\ K^{-7}$
**Olivine Radiative Thermal Conductivity – Ref.[5]** - *Shankland et al. (1979, JGR)*		
$\boldsymbol{T\ [K]}$		$\boldsymbol{\Lambda_{rad}\ [W\ m^{-1}\ K^{-1}]}$
**c-axis**		
796.52		0.84 (-0.20; +0.07)
1666.09		1.99 (-0.11; +0.04)
**a-axis**		
796.52		1.51 (-0.54; +0.35)
1071.30		1.70 (-0.27; +0.20)
1366.96		2.02 (-0.18; +0.08)
1666.09		2.21 (-0.11; +0.05)

Extended Data Tab. 3 | List of parameters used to compute the lattice and the radiative thermal conductivity in the extended equations (S10) and (S12). The value of the refractive index $n = 1.6$, is a direction average value for peridotitic olivine at 1 atm. The values for total thermal conductivity were taken from *Hofmeister (1999, Science)*[6], table 1 - line Olivine $Fo_{90}Fa_{10}$. The radiative thermal conductivity data were extracted with WebPlotDigitizer (https://apps.automeris.io/wpd/) from: *Shankland et al. (1979, JGR)*[5] (see Figure 6 in their paper); and from *Grose & Afonso (2019, G-Cubed)*[8] (see Figure 6: olivine, 1 cm mean grain radius curve in red). The values in the brackets represent the uncertainties of Λ_{rad} reported by Ref.[5].

Cell Volume Coefficients - V_{cell}^{x} [Å^3]						
		a^x [$Å^3\ GPa^{-4}$]	b^x [$Å^3\ GPa^{-3}$]	c^x [$Å^3\ GPa^{-2}$]	d^x [$Å^3\ GPa^{-1}$]	V_0^x [Å^3]
**Ol**	**Ref.[38]**	6.3936×10^{-4}	-2.4866×10^{-2}	3.3457×10^{-1}	-3.4187	290.18
**Rw**	**Ref.[39]**	2.3973×10^{-5}	-1.7392×10^{-3}	7.2840×10^{-2}	-3.3635	528.75
**Thermal Expansion Coefficient - α^x [K^{-1}]**						
		e^{Ol} [K^{-2}]	f^{Ol} [$K^{-1}\ GPa^{-1}$]	g^{Ol} [$K^{-2}\ GPa^{-1}$]	h^{Ol} [$K^{-1}\ GPa^{-2}$]	α_0^x [K^{-1}]
**Ol**	**Ref.[41]**	1.695×10^{-8}	-1.030×10^{-6}	-7.907×10^{-10}	2.286×10^{-8}	2.627×10^{-5}
**Rw**	**Ref.[42]**	i_y^{Rw}	j_y^{Rw}	k_y^{Rw}	l_y^{Rw}	m_y^{Rw}
Q_α [$K^{-1}\ GPa^{-2}$]		-1.9189×10^{-21} [$K^{-1}\ GPa^{-2}$]	1.0264×10^{-17} [$K^{-1}\ GPa^{-2}$]	-1.8113×10^{-14} [$K^{-1}\ GPa^{-2}$]	1.7121×10^{-11} [$K^{-1}\ GPa^{-2}$]	2.8386×10^{-9} [$K^{-1}\ GPa^{-2}$]
L_α [$K^{-1}\ GPa^{-1}$]		1.7149×10^{-19} [$K^{-1}\ GPa^{-1}$]	-9.0381×10^{-16} [$K^{-1}\ GPa^{-1}$]	1.6396×10^{-12} [$K^{-1}\ GPa^{-1}$]	-1.4652×10^{-9} [$K^{-1}\ GPa^{-1}$]	-1.5839×10^{-7} [$K^{-1}\ GPa^{-1}$]
I_α [K^{-1}]		-1.0115×10^{-17} [K^{-1}]	5.3389×10^{-14} [K^{-1}]	-1.0089×10^{-10} [K^{-1}]	8.7225×10^{-8} [K^{-1}]	4.6176×10^{-7} [K^{-1}]
**Specific Heat Capacity Coefficients - Cp_{Sp}^x [$J\ kg^{-1}K^{-1}$]**						
		n^{Ol} [$J\ kg^{-1}K^{-0.5}$]	o^{Ol} [$J\ K\ kg^{-1}$]	p^{Ol} [$J\ kg^{-1}K^{-1}\ GPa^{-1}$]	q^{Ol} [$J\ kg^{-1}K^{-1}\ GPa^{-2}$]	Cp_0^x [$J\ kg^{-1}K^{-1}$]
**Ol**	**Ref.[41]**	-1.238×10^{4}	-3.139×10^{6}	-3.184	8.414×10^{-2}	1585
**Molar Heat Capacity Coefficients - Cp_{mol}^x [$J\ mol^{-1}K^{-1}$]**						
**Rw**	**Ref.[42]**	r_y^{Rw}	s_y^{Rw}	t_y^{Rw}	u_y^{Rw}	v_y^{Rw}
Q_α [$J\ mol^{-1}K^{-1}\ GPa^{-2}$]		3.7084×10^{-15} [$J\ mol^{-1}K^{-5}\ GPa^{-2}$]	-1.9040×10^{-11} [$J\ mol^{-1}K^{-4}\ GPa^{-2}$]	3.7691×10^{-8} [$J\ mol^{-1}K^{-3}\ GPa^{-2}$]	-2.9088×10^{-5} [$J\ mol^{-1}K^{-2}\ GPa^{-2}$]	1.0283×10^{-2} [$J\ mol^{-1}K^{-1}\ GPa^{-2}$]
L_α [$J\ mol^{-1}K^{-1}\ GPa^{-1}$]		-2.4502×10^{-13} [$J\ mol^{-1}K^{-5}\ GPa^{-1}$]	1.3509×10^{-9} [$J\ mol^{-1}K^{-4}\ GPa^{-1}$]	-2.8953×10^{-6} [$J\ mol^{-1}K^{-3}\ GPa^{-1}$]	2.5867×10^{-3} [$J\ mol^{-1}K^{-2}\ GPa^{-1}$]	-1.0847 [$J\ mol^{-1}K^{-1}\ GPa^{-1}$]
I_α [$J\ mol^{-1}K^{-1}$]		-6.4406×10^{-11} [$J\ mol^{-1}K^{-5}$]	3.3921×10^{-7} [$J\ mol^{-1}K^{-4}$]	-6.4394×10^{-4} [$J\ mol^{-1}K^{-3}$]	5.3896×10^{-1} [$J\ mol^{-1}K^{-2}$]	4.1003 [$J\ mol^{-1}K^{-1}$]

Extended Data Tab. 4 | List of parameters used to compute: unit cell volume V_{cell}^x [Å^3] eq. (S13); molar volume V_{mol}^x [$cm^3 mol^{-1}$] eq. (S14); thermal expansion coefficient $\alpha^x [K^{-1}]$ eq. (S17); specific heat capacity Cp_{Sp}^x [$J\ kg^{-1}K^{-1}$] eq. (S20); and molar heat capacity Cp_{mol}^x [$J\ mol^{-1}K^{-1}$] eq. (S21)-(S22).

Symbol	Parameter	Value	Unit
η_0	Reference viscosity	1.00×10^{20}	$[Pa\ s]$
T_0	Reference temperature	1600	[K]
E	Activation energy	3.0×10^{5}	$[J\ mol^{-1}]$
V	Activation volume	5.0×10^{-6}	$[m^3\ mol^{-1}]$
R	Perfect gas constant	8.314	$[J\ mol^{-1}\ \mathrm{K}^{-1}]$

Extended Data Tab. 5 | Parameters to compute viscosity with the Arrhenius Law, see eq. (S32).

Phase	Chemical Formula
**Olivine Polymorphs**	
Forsterite	$\mathrm{Mg_2SiO_4}$
Dry Wadsleyite	$\mathrm{Mg_2SiO_4}$
Dry Ringwoodite	$\mathrm{Mg_2SiO_4}$
Wet Wadsleyite	$\mathrm{Mg_{1.89}Si_{0.98}O_{3.7}(OH)_{0.3}}$
Wet Ringwoodite	$\mathrm{Mg_{1.89}Si_{0.98}O_{3.7}(OH)_{0.3}}$
**Pyroxene Polymorphs**	
Enstatite	$\mathrm{MgSiO_3}$
Akimotoite	$\mathrm{MgSiO_3}$
Bridgmanite	$\mathrm{MgSiO_3}$
**Serpentine Polymorphs**	
Antigorite	$\mathrm{Mg_{48}Si_{34}O_{85}(OH)_{62}}$
Phase A	$\mathrm{Mg_7Si_2O_8(OH)_6}$
Phase E	$\mathrm{Mg_{2.30}Si_{1.28}O_{3.65}(OH)_{2.42}}$
Super-hydrous Phase B	$\mathrm{Mg_{10}Si_3O_{14}(OH)_4}$
Phase B	$\mathrm{Mg_{12}Si_4O_{19}(OH)_2}$
Anhydrous Phase B	$\mathrm{Mg_{14}Si_5O_{24}}$
Phase D	$\mathrm{MgSi_2O_4(OH)_2}$
**Talc Polymorph**	
Talc	$\mathrm{Mg_3Si_4O_{10}(OH)_2}$
Phase 10Å	$\mathrm{Mg_3Si_4O_{14}H_6}$
**Humite Group**	
Humite	$\mathrm{Mg_7(SiO_4)_3(OH)_2}$
Clinohumite	$\mathrm{Mg_9Si_4O_{16}(OH)_2}$
Chondrodite	$\mathrm{Mg_5(SiO_4)_2(OH)_2}$
**Hydrous Oxides**	
Brucite	$\mathrm{Mg(OH)_2}$
**Oxides**	
Periclase	MgO
Stishovite	$\mathrm{SiO_2}$
Water	$\mathrm{H_2O}$

Extended Data Tab. 6 | Phases of the MgO-SiO_2-H_2O (MSH) system and their chemical formulas[26].

		**20** $Myrs$	**40** $Myrs$	**60** $Myrs$	**80** $Myrs$
**2.5** $cm\ yr^{-1}$	$\Lambda_{rad} = 0$	1379	1288	1248	1226
	$\Lambda_{rad} > 0$	1517	1421	1375	1349
**5.0** $cm\ yr^{-1}$	$\Lambda_{rad} = 0$	1249	1159	1118	1093
	$\Lambda_{rad} > 0$	1378	1279	1235	1208
**7.5** $cm\ yr^{-1}$	$\Lambda_{rad} = 0$	1185	1092	1049	1022
	$\Lambda_{rad} > 0$	1301	1202	1156	1128
**10.5** $cm\ yr^{-1}$	$\Lambda_{rad} = 0$	1146	1049	1003	975
	$\Lambda_{rad} > 0$	1251	1150	1103	1074
**12.5** $cm\ yr^{-1}$	$\Lambda_{rad} = 0$	1118	1018	971	942
	$\Lambda_{rad} > 0$	1215	1113	1065	1034

Extended Data Tab. 7 | *rms* temperature T_{rms} $[K]$ of the upper 40 km of the slab. In each cell, the top value belongs to the model set 1 ($\Lambda_{rad} = 0$), whereas the bottom value belongs to the model set 2 ($\Lambda_{rad} > 0$).

		**20** $Myrs$		**40** $Myrs$		**60** $Myrs$		**80** $Myrs$	
		10 km	30 km	10 km	30 km	10 km	30 km	10 km	30 km
**2.5** $cm\ yr^{-1}$	$\Lambda_{rad} = 0$	117.9	60.0	141.4	60.0	161.4	660.9	185.4	660.9
	$\Lambda_{rad} > 0$	87.4	60.0	109.9	60.0	129.9	660.9	149.9	660.9
**5.0** $cm\ yr^{-1}$	$\Lambda_{rad} = 0$	155.9	60.0	200.4	60.0	660.9	660.9	660.9	660.9
	$\Lambda_{rad} > 0$	111.9	60.0	135.9	60.0	157.4	660.9	179.4	660.9
**7.5** $cm\ yr^{-1}$	$\Lambda_{rad} = 0$	206.4	60.0	660.9	60.0	660.9	660.9	660.9	660.9
	$\Lambda_{rad} > 0$	132.9	60.0	159.9	60.0	189.4	660.9	230.9	660.9
**10.5** $cm\ yr^{-1}$	$\Lambda_{rad} = 0$	660.9	60.0	660.9	60.0	660.9	660.9	660.9	660.9
	$\Lambda_{rad} > 0$	151.9	60.0	192.4	60.0	273.4	660.9	660.9	660.9
**12.5** $cm\ yr^{-1}$	$\Lambda_{rad} = 0$	660.9	60.0	660.9	60.0	660.9	660.9	660.9	660.9
	$\Lambda_{rad} > 0$	169.9	60.0	660.9	60.0	660.9	660.9	660.9	660.9

Extended Data Tab. 8 | Dehydration depth in the mantle D_{dhy} $[km]$ at 10 km and 30 km inside the slab. In each cell, the top value belongs to the model set 1 ($\Lambda_{rad} = 0$), whereas the bottom value belongs to the model set 2 ($\Lambda_{rad} > 0$).

		$20\ Myrs$	$40\ Myrs$	$60\ Myrs$	$80\ Myrs$
$2.5\ cm\ yr^{-1}$	$\Lambda_{rad} = 0$	1.67×10^{23}	4.15×10^{24}	2.26×10^{25}	6.86×10^{25}
	$\Lambda_{rad} > 0$	4.95×10^{21}	9.06×10^{22}	4.54×10^{23}	1.30×10^{24}
$5.0\ cm\ yr^{-1}$	$\Lambda_{rad} = 0$	3.16×10^{24}	1.41×10^{26}	1.28×10^{27}	6.19×10^{27}
	$\Lambda_{rad} > 0$	9.20×10^{22}	3.21×10^{24}	2.59×10^{25}	1.17×10^{26}
$7.5\ cm\ yr^{-1}$	$\Lambda_{rad} = 0$	1.57×10^{25}	1.10×10^{27}	1.43×10^{28}	9.33×10^{28}
	$\Lambda_{rad} > 0$	5.29×10^{23}	2.90×10^{25}	3.34×10^{26}	2.05×10^{27}
$10.5\ cm\ yr^{-1}$	$\Lambda_{rad} = 0$	4.69×10^{25}	4.63×10^{27}	7.85×10^{28}	6.29×10^{29}
	$\Lambda_{rad} > 0$	1.83×10^{24}	1.42×10^{26}	2.14×10^{27}	1.64×10^{28}
$12.5\ cm\ yr^{-1}$	$\Lambda_{rad} = 0$	1.07×10^{26}	1.39×10^{28}	2.88×10^{29}	2.68×10^{30}
	$\Lambda_{rad} > 0$	4.76×10^{24}	4.85×10^{26}	9.05×10^{27}	8.20×10^{28}

Extended Data Tab. 9 | *rms* viscosity η_{rms} $[Pa\ s]$ of the upper 40 km of the slab. In each cell, the top value belongs to the model set 1 ($\Lambda_{rad} = 0$), whereas the bottom value belongs to the model set 2 ($\Lambda_{rad} > 0$).

		$20\ Myrs$	$40\ Myrs$	$60\ Myrs$	$80\ Myrs$
$2.5\ cm\ yr^{-1}$	$\Lambda_{rad} = 0$	3501	3524	3540	3554
	$\Lambda_{rad} > 0$	3482	3507	3524	3539
$5.0\ cm\ yr^{-1}$	$\Lambda_{rad} = 0$	3516	3538	3554	3568
	$\Lambda_{rad} > 0$	3500	3524	3540	3555
$7.5\ cm\ yr^{-1}$	$\Lambda_{rad} = 0$	3522	3545	3561	3575
	$\Lambda_{rad} > 0$	3508	3532	3549	3564
$10.5\ cm\ yr^{-1}$	$\Lambda_{rad} = 0$	3526	3549	3565	3580
	$\Lambda_{rad} > 0$	3514	3538	3555	3570
$12.5\ cm\ yr^{-1}$	$\Lambda_{rad} = 0$	3529	3552	3568	3583
	$\Lambda_{rad} > 0$	3518	3542	3559	3574

Extended Data Tab. 10 | *rms* density ρ_{rms} $[kg\ m^{-3}]$ of the upper 40 km of the slab. In each cell, the top value belongs to the model set 1 ($\Lambda_{rad} = 0$), whereas the bottom value belongs to the model set 2 ($\Lambda_{rad} > 0$).

		$20\ Myrs$		$40\ Myrs$		$60\ Myrs$		$80\ Myrs$	
		10 km	30 km	10 km	30 km	10 km	30 km	10 km	30 km
$\mathbf{2.5}$	$\Lambda_{rad} = 0$	143.9	194.4	168.9	276.9	189.4	323.9	208.4	358.9
$cm\ yr^{-1}$	$\Lambda_{rad} > 0$	99.4	136.4	121.4	208.4	141.9	250.4	161.9	281.9
$\mathbf{5.0}$	$\Lambda_{rad} = 0$	195.9	201.9	227.4	363.9	251.4	446.4	271.4	510.9
$cm\ yr^{-1}$	$\Lambda_{rad} > 0$	131.9	60.0	156.9	277.9	178.9	343.4	198.9	389.4
$\mathbf{7.5}$	$\Lambda_{rad} = 0$	237.4	60.0	276.4	438.4	302.9	559.9	324.9	610.4
$cm\ yr^{-1}$	$\Lambda_{rad} > 0$	159.9	60.0	188.9	333.9	212.4	428.9	233.9	514.9
$\mathbf{10.5}$	$\Lambda_{rad} = 0$	274.4	60.0	318.9	511.9	348.4	617.9	371.9	627.9
$cm\ yr^{-1}$	$\Lambda_{rad} > 0$	185.9	60.0	219.4	382.9	244.4	530.4	266.4	607.4
$\mathbf{12.5}$	$\Lambda_{rad} = 0$	306.9	60.0	357.4	575.9	389.4	629.4	415.9	634.9
$cm\ yr^{-1}$	$\Lambda_{rad} > 0$	210.4	60.0	247.9	437.9	274.9	607.4	297.9	624.9

Extended Data Tab. 11 | *max* depth of $650°C$ isotherm D_{650} $[km]$ inside the slab. In each cell, the top value belongs to the model set 1 ($\Lambda_{rad} = 0$), whereas the bottom value belongs to the model set 2 ($\Lambda_{rad} > 0$).

		$20\ Myrs$		$40\ Myrs$		$60\ Myrs$		$80\ Myrs$	
		10 km	30 km	10 km	30 km	10 km	30 km	10 km	30 km
$\mathbf{2.5}$	$\Lambda_{rad} = 0$	426.4	415.4	421.9	404.9	419.9	400.4	417.9	396.9
$cm\ yr^{-1}$	$\Lambda_{rad} > 0$	433.9	426.9	430.4	416.4	428.4	410.4	426.9	406.9
$\mathbf{5.0}$	$\Lambda_{rad} = 0$	416.4	404.4	412.4	395.4	409.9	390.9	407.9	390.9
$cm\ yr^{-1}$	$\Lambda_{rad} > 0$	425.9	414.4	422.4	403.4	420.4	397.9	418.9	393.9
$\mathbf{7.5}$	$\Lambda_{rad} = 0$	410.4	400.9	405.9	391.9	403.4	387.4	400.9	384.4
$cm\ yr^{-1}$	$\Lambda_{rad} > 0$	420.9	407.9	416.9	397.4	413.9	390.9	412.9	388.4
$\mathbf{10.5}$	$\Lambda_{rad} = 0$	405.4	399.4	400.9	390.4	397.9	384.9	395.9	382.9
$cm\ yr^{-1}$	$\Lambda_{rad} > 0$	416.4	404.4	412.4	394.4	410.4	388.9	408.4	385.4
$\mathbf{12.5}$	$\Lambda_{rad} = 0$	401.9	398.9	396.9	389.9	393.9	384.9	390.9	381.9
$cm\ yr^{-1}$	$\Lambda_{rad} > 0$	412.9	402.9	408.9	392.4	406.4	387.4	403.4	383.9

Extended Data Tab. 12 | Depth of olivine-wadsleyite reaction D_{ol} $[km]$ at 10 km inside the slab. In each cell, the top value belongs to the model set 1 ($\Lambda_{rad} = 0$), whereas the bottom value belongs to the model set 2 ($\Lambda_{rad} > 0$). The Clapeyron slope is 1.94 MPa/K Ref.[26] *Komabayashi et al., (2004, JGR).*

		**20 $Myrs$**		**40 $Myrs$**		**60 $Myrs$**		**80 $Myrs$**	
		10 km	30 km	10 km	30 km	10 km	30 km	10 km	30 km
**2.5 $cm\ yr^{-1}$**	$\Lambda_{rad} = 0$	0.0	0.0	0.0	0.0	0.0	0.0	0.0	0.0
	$\Lambda_{rad} > 0$	0.0	0.0	0.0	0.0	0.0	0.0	0.0	0.0
**5.0 $cm\ yr^{-1}$**	$\Lambda_{rad} = 0$	0.0	0.0	0.0	0.0	0.0	55.5	0.0	123.0
	$\Lambda_{rad} > 0$	0.0	0.0	0.0	0.0	0.0	0.0	0.0	0.0
**7.5 $cm\ yr^{-1}$**	$\Lambda_{rad} = 0$	0.0	0.0	0.0	46.5	0.0	172.5	0.0	226.0
	$\Lambda_{rad} > 0$	0.0	0.0	0.0	0.0	0.0	38.0	0.0	126.5
**10.5 $cm\ yr^{-1}$**	$\Lambda_{rad} = 0$	0.0	0.0	0.0	121.5	0.0	233.0	0.0	245.0
	$\Lambda_{rad} > 0$	0.0	0.0	0.0	0.0	0.0	141.5	0.0	222.0
**12.5 $cm\ yr^{-1}$**	$\Lambda_{rad} = 0$	0.0	0.0	0.0	186.0	0.0	244.5	25.0	253.0
	$\Lambda_{rad} > 0$	0.0	0.0	0.0	45.5	0.0	220.0	0.0	241.0

Extended Data Tab. 13 | Length of the metastable olivine wedge[60] L_{ol} $[km]$ at 10 km inside the slab. In each cell, the top value belongs to the model set 1 ($\Lambda_{rad} = 0$), whereas the bottom value belongs to the model set 2 ($\Lambda_{rad} > 0$). This parameter represents the difference between the maximum depth of $650°C$ isotherm $D_{650}[km]$ and the depth of olivine-wadsleyite reaction D_{ol} $[km]$: $L_{ol} = D_{650} - D_{ol}$. When the difference is negative, L_{ol} is set to 0.

References of Supplementary

1. **Ishii, T., Kojitani, H., & Akaogi, M. (2011).** Post-spinel transitions in pyrolite and Mg_2SiO_4 and akimotoite–perovskite transition in $MgSiO_3$: precise comparison by high-pressure high-temperature experiments with multi-sample cell technique. *Earth and Planetary Science Letters*, 309(3-4), 185-197. https://doi.org/10.1016/j.epsl.2011.06.023
2. **Lindsley, Donald H., ed. (1991).** Oxide Minerals: Petrologic and magnetic significance. *Reviews in Mineralogy. Vol. 25. Washington (D.C.): Mineralogical Society of America*. p. 509. ISBN 0-939950-30-8.
3. **Lambart, S., Hamilton, S., & Lang, O. I. (2022).** Compositional variability of San Carlos olivine. *Chemical Geology*, 605, 120968. https://doi.org/10.1016/j.chemgeo.2022.120968
4. **Smith, H. G., & Langer, K. (1982).** Single crystal spectra of olivines in the range 40,000-5,000 cm− 1 at pressures up to 200 kbar. *American Mineralogist*, 67(3-4), 343-348.
5. **Shankland, T. J., Nitsan, U., & Duba, A. G. (1979).** Optical absorption and radiative heat transport in olivine at high temperature. *Journal of Geophysical Research: Solid Earth*, 84(B4), 1603-1610. https://doi.org/10.1029/JB084iB04p01603
6. **Hofmeister, A. M. (1999).** Mantle values of thermal conductivity and the geotherm from phonon lifetimes. *Science*, 283(5408), 1699-1706. https://doi.org/10.1126/science.283.5408.1699
7. **Ullrich, K., Langer, K., & Becker, K. D. (2002).** Temperature dependence of the polarized electronic absorption spectra of olivines. Part I–fayalite. *Physics and Chemistry of Minerals*, 29, 409-419. https://doi.org/10.1007/s00269-002-0248-4
8. **Grose, C. J., & Afonso, J. C. (2019).** New constraints on the thermal conductivity of the upper mantle from numerical models of radiation transport. *Geochemistry, Geophysics, Geosystems (G-Cubed)*, 20(5), 2378-2394. https://doi.org/10.1029/2019GC008187
9. **Lobanov, S. S., Schifferle, L., & Schulz, R. (2020).** Gated detection of supercontinuum pulses enables optical probing of solid and molten silicates at extreme pressure–temperature conditions. *Review of Scientific Instruments*, 91(5). https://doi.org/10.1063/5.0004590
10. **Lobanov, S. S., & Geballe, Z. M. (2022).** Non-isotropic contraction and expansion of samples in diamond anvil cells: Implications for thermal conductivity at the core-mantle boundary. *Geophysical Research Letters*, 49(19), e2022GL100379. https://doi.org/10.1029/2022GL100379
11. **Speziale, S., Duffy, T. S., & Angel, R. J. (2004).** Single-crystal elasticity of fayalite to 12 GPa. *Journal of Geophysical Research: Solid Earth*, 109(B12). https://doi.org/10.1029/2004JB003162
12. **Hofmeister, A. M. (2005).** Dependence of diffusive radiative transfer on grain-size, temperature, and Fe-content: Implications for mantle processes. *Journal of Geodynamics*, 40(1), 51-72. https://doi.org/10.1016/j.jog.2005.06.001
13. **Keppler, H., & Smyth, J. R. (2005).** Optical and near infrared spectra of ringwoodite to 21.5 GPa: Implications for radiative heat transport in the mantle. *American Mineralogist*, 90(7), 1209-1212. https://doi.org/10.2138/am.2005.1908
14. **Lobanov, S. S., Holtgrewe, N., Ito, G., Badro, J., Piet, H., Nabiei, F., ... & Goncharov, A. F. (2020).** Blocked radiative heat transport in the hot pyrolitic lower mantle. *Earth and Planetary Science Letters*, 537, 116176. https://doi.org/10.1016/j.epsl.2020.116176
15. **Lobanov, S. S., Soubiran, F., Holtgrewe, N., Badro, J., Lin, J. F., & Goncharov, A. F. (2021).** Contrasting opacity of bridgmanite and ferropericlase in the lowermost mantle:

Implications to radiative and electrical conductivity. *Earth and Planetary Science Letters*, 562, 116871. https://doi.org/10.1016/j.epsl.2021.116871

16. **Fabian, D., Henning, T., Jäger, C., Mutschke, H., Dorschner, J., & Wehrhan, O. (2001).** Steps toward interstellar silicate mineralogy-VI. Dependence of crystalline olivine IR spectra on iron content and particle shape. *Astronomy & Astrophysics*, 378(1), 228-238. https://doi.org/10.1051/0004-6361:20011196
17. **Zeidler, S., Posch, T., Mutschke, H., Richter, H., & Wehrhan, O. (2011).** Near-infrared absorption properties of oxygen-rich stardust analogs-the influence of coloring metal ions. *Astronomy & Astrophysics*, 526, A68. https://doi.org/10.1051/0004-6361/201015219
18. **Zeidler, S., Mutschke, H., & Posch, T. (2015).** Temperature-dependent infrared optical constants of olivine and enstatite. *The Astrophysical Journal*, 798(2), 125. https://doi.org/10.1088/0004-637X/798/2/125
19. **Thomas, S. M., Bina, C. R., Jacobsen, S. D., & Goncharov, A. F. (2012).** Radiative heat transfer in a hydrous mantle transition zone. *Earth and Planetary Science Letters*, 357, 130-136. https://doi.org/10.1016/j.epsl.2012.09.035
20. **Katsura, T. (2022).** A revised adiabatic temperature profile for the mantle. *Journal of Geophysical Research: Solid Earth*, 127(2), e2021JB023562. https://doi.org/10.1029/2021JB023562
21. **Chang, Y. Y., Hsieh, W. P., Tan, E., & Chen, J. (2017)**. Hydration-reduced lattice thermal conductivity of olivine in Earth's upper mantle. *Proceedings of the National Academy of Sciences*, 114(16), 4078-4081. https://doi.org/10.1073/pnas.1616216114
22. **Marzotto, E., Hsieh, W. P., Ishii, T., Chao, K. H., Golabek, G. J., Thielmann, M., & Ohtani, E. (2020).** Effect of water on lattice thermal conductivity of ringwoodite and its implications for the thermal evolution of descending slabs. *Geophysical Research Letters*, 47(13), e2020GL087607. https://doi.org/10.1029/2020GL087607
23. **Xu, Y., Shankland, T. J., Linhardt, S., Rubie, D. C., Langenhorst, F., & Klasinski, K. (2004).** Thermal diffusivity and conductivity of olivine, wadsleyite and ringwoodite to 20 GPa and 1373 K. *Physics of the Earth and Planetary Interiors*, 143, 321-336. https://doi.org/10.1016/j.pepi.2004.03.005
24. **Dubuffet, F., Yuen, D. A., & Rabinowicz, M. (1999).** Effects of a realistic mantle thermal conductivity on the patterns of 3-D convection. *Earth and Planetary Science Letters*, 171(3), 401-409. https://doi.org/10.1016/S0012-821X(99)00165-X
25. **Dziewonski, A. M., & Anderson, D. L. (1981).** Preliminary reference Earth model. *Physics of the Earth and Planetary Interiors*, 25(4), 297-356. https://doi.org/10.1016/0031-9201(81)90046-7
26. **Komabayashi, T., Omori, S., & Maruyama, S. (2004).** Petrogenetic grid in the system MgO-SiO_2-H_2O up to 30 GPa, 1600 C: Applications to hydrous peridotite subducting into the Earth's deep interior. *Journal of Geophysical Research: Solid Earth*, 109(B3). https://doi.org/10.1029/2003JB002651

27. **Marquardt, H., Ganschow, S., & Schilling, F. R. (2009).** Thermal diffusivity of natural and synthetic garnet solid solution series. *Physics and Chemistry of Minerals*, *36*, 107-118. https://doi.org/10.1007/s00269-008-0261-3
28. **Klemens, P. G. (1994).** Phonon scattering and thermal resistance due to grain boundaries. *International Journal of Thermophysics*, 15, 1345-1351. https://doi.org/10.1007/BF01458842
29. **Birch, F., & Clark, H. (1940).** The thermal conductivity of rocks and its dependence upon temperature and composition; Part II. *American Journal of Science*, 238(9), 613-635. https://doi.org/10.2475/ajs.238.9.613
30. **Lobanov, S. S., Speziale, S., & Brune, S. (2021).** Modelling Mie scattering in pyrolite in the laser-heated diamond anvil cell: Implications for the core-mantle boundary temperature determination. *Physics of the Earth and Planetary Interiors*, 318, 106773. https://doi.org/10.1016/j.pepi.2021.106773
31. **Powell, R. W., & Griffiths, E. (1937).** The variation with temperature of the thermal conductivity and the X-ray structure of some micas I—The thermal conductivity up to 600° C. *Proceedings of the Royal Society of London. Series A-Mathematical and Physical Sciences*, 163(913), 189-198. https://doi.org/10.1098/rspa.1937.0220
32. **Wood, W. A. (1937).** The variation with temperature of the thermal conductivity and the X-ray structure of some micas-II—The X-ray examination of the structure. *Proceedings of the Royal Society of London. Series A-Mathematical and Physical Sciences*, 163(913), 199-204. https://doi.org/10.1098/rspa.1937.0221
33. **Karato, S. I., & Wu, P. (1993).** Rheology of the upper mantle: A synthesis. *Science*, 260(5109), 771-778. https://doi.org/10.1126/science.260.5109.771
34. **Hirth, G., & Kohlstedf, D. (2003).** Rheology of the upper mantle and the mantle wedge: A view from the experimentalists. *Geophysical Monograph-American Geophysical Union*, 138, 83-106. https://doi.org/10.1029/138GM06
35. **Faul, U. H., & Jackson, I. (2005).** The seismological signature of temperature and grain size variations in the upper mantle. *Earth and Planetary Science Letters*, 234(1-2), 119-134. https://doi.org/10.1016/j.epsl.2005.02.008
36. **Armienti, P., & Tarquini, S. (2002).** Power law olivine crystal size distributions in lithospheric mantle xenoliths. *Lithos*, 65(3-4), 273-285. https://doi.org/10.1016/S0024-4937(02)00195-0
37. **Branlund, J. M., Kameyama, M. C., Yuen, D. A., & Kaneda, Y. (2000).** Effects of temperature-dependent thermal diffusivity on shear instability in a viscoelastic zone: implications for faster ductile faulting and earthquakes in the spinel stability field. *Earth and Planetary Science Letters*, 182(2), 171-185. https://doi.org/10.1016/S0012-821X(00)00239-9
38. **Downs, R. T., Zha, C. S., Duffy, T. S., & Finger, L. W. (1996).** The equation of state of forsterite to 17.2 GPa and effects of pressure media. *American Mineralogist*, 81(1–2), 51–55. https://doi.org/10.2138/am-1996-1-207
39. **Ye, Y., Brown, D. a., Smyth, J. R., Panero, W. R., Jacobsen, S. D., Chang, Y.-Y., et al. (2012).** Compressibility and thermal expansion of hydrous ringwoodite with 2.5(3) wt% H_2O. *American Mineralogist*, 97(4), 573–582. https://doi.org/10.2138/am.2012.4010
40. **Cloetingh, S., Koptev, A., Lavecchia, A., Kovács, I. J., & Beekman, F. (2022).** Fingerprinting secondary mantle plumes. *Earth and Planetary Science Letters*, *597*, 117819. https://doi.org/10.1016/j.epsl.2022.117819
41. **Su, C., Liu, Y., Song, W., Fan, D., & Wang, Z. (2018).** Thermodynamic properties of San Carlos olivine at high temperature and high pressure. *Acta Geochimica*, 37(2), 171–179.

https://doi.org/10.1007/s11631-018-0261-z
42. **Dorogokupets, P. I., Dymshits, A. M., Sokolova, T. S., Danilov, B. S., & Litasov, K. D. (2015).** The equations of state of forsterite, wadsleyite, ringwoodite, akimotoite, $MgSiO_3$-perovskite, and postperovskite and phase diagram for the Mg_2SiO_4 system at pressures of up to 130 GPa. *Russian Geology and Geophysics*, 56(1–2), 172–189. https://doi.org/10.1016/j.rgg.2015.01.011
43. **Turcotte, D. L. & Schubert, G. (2014).** Geodynamics (3rd ed.). *Cambridge: Cambridge University Press*. https://doi.org/10.1017/CBO9780511843877
44. **Stein C. A., & Stein, S. (1992).** A model for the global variation in oceanic depth and heat flow with lithospheric age. *Nature*, 359(6391), 123–129. https://doi.org/10.1038/359123a0
45. **Crameri, F., Shephard, G. E., & Heron, P. J. (2020).** The misuse of color in science communication. *Nature Communication*, 11(1), 5444. https://doi.org/10.1038/s41467-020-19160-7
46. **Clauser, C., & Huenges, E. (1995).** Thermal conductivity of rocks and minerals. In: Rock physics and phase relations: a handbook of physical constants, T.J. Ahrens (Ed.), 3-9, 105-126. https://doi.org/10.1029/RF003p0105
47. **Hearn, E. H., Humphreys, E. D., Chai, M., & Brown, J. M. (1997).** Effect of anisotropy on oceanic upper mantle temperatures, structure, and dynamics. *Journal of Geophysical Research: Solid Earth*, 102(B6), 11943-11956. https://doi.org/10.1029/97JB00506
48. **Chai, M., Brown, J. M., & Slutsky, L. J. (1996).** Thermal diffusivity of mantle minerals. *Physics and Chemistry of Minerals*, 23, 470-475. https://doi.org/10.1007/BF00202033
49. **Gerya, T. (2019).** Introduction to Numerical Geodynamic Modelling (2nd Ed.). *Cambridge University Press*, 484 pages. https://doi.org/10.1017/9781316534243
50. **Chien, Y. H., Marzotto, E., Tsao, Y. C., & Hsieh, W. P. (2024).** Anisotropic thermal conductivity of antigorite along slab subduction impacts seismicity of intermediate-depth earthquakes. *Nature Communications*, 15(1), 5198. https://doi.org/10.1038/s41467-024-49418-3
51. **Van Keken, P. E., Hacker, B. R., Syracuse, E. M., & Abers, G. A. (2011).** Subduction factory: 4. Depth-dependent flux of H_2O from subducting slabs worldwide. *Journal of Geophysical Research: Solid Earth*, 116(B1). https://doi.org/10.1029/2010JB007922
52. **Syracuse, E. M., van Keken, P. E., & Abers, G. A. (2010).** The global range of subduction zone thermal models. *Physics of the Earth and Planetary Interiors*, 183(1-2), 73-90. https://doi.org/10.1016/j.pepi.2010.02.004
53. **Weertman, J. (1970).** The creep strength of the Earth's mantle. *Reviews of Geophysics*, 8(1), 145-168. https://doi.org/10.1029/RG008i001p00145
54. **Stixrude, L., & Lithgow-Bertelloni, C. (2005).** Thermodynamics of mantle minerals—I. Physical properties. *Geophysical Journal International*, 162(2), 610-632. https://doi.org/10.1111/j.1365-246X.2005.02642.x
55. **Irifune, T., & Ringwood, A. E. (1987).** Phase transformations in a harzburgite composition to 26 GPa: implications for dynamical behaviour of the subducting slab. *Earth and Planetary Science Letters*, 86(2-4), 365-376. https://doi.org/10.1016/0012-821X(87)90233-0
56. **Faccenda, M., Gerya, T. V., Mancktelow, N. S., & Moresi, L. (2012)**. Fluid flow during slab unbending and dehydration: Implications for intermediate-depth seismicity, slab weakening and deep-water recycling. *Geochemistry, Geophysics, Geosystems (G-cubed)*, 13(1). https://doi.org/10.1029/2011GC003860

57. **Ohtani, E. (2021).** Hydration and dehydration in Earth's interior. *Annual Review of Earth and Planetary Sciences*, 49(1), 253-278. https://doi.org/10.1146/annurev-earth-080320-062509
58. **Horiuchi, Hiroyuki, Morimoto, Nobuo, Yamamoto, K., & Akimoto, S. I. (1979).** Crystal structure of $2Mg_2SiO_4 \cdot 3Mg(OH)_2$, a new high-pressure structure type. *American Mineralogist*, 64(5-6), 593-598.
59. **Faccenda, M. (2014).** Water in the slab: A trilogy. *Tectonophysics*, 614, 1-30. https://doi.org/10.1016/j.tecto.2013.12.020
60. **Ishii, T., & Ohtani, E. (2021).** Dry metastable olivine and slab deformation in a wet subducting slab. *Nature Geoscience*, *14*(7), 526-530. https://doi.org/10.1038/s41561-021-00756-7
